# RNA–DNA Differences: Mechanisms, Oxidative Stress, Transcriptional Fidelity, and Health Implications

**DOI:** 10.3390/antiox14050544

**Published:** 2025-04-30

**Authors:** Viktor Stolc, Ondrej Preto, Miloslav Karhanek, Friedemann Freund, Yuri Griko, David J. Loftus, Maurice M. Ohayon

**Affiliations:** 1 NASA Ames Research Center, Moffett Field, CA 94035, USA; 2 Biomedical Research Center, Slovak Academy of Sciences, 845 05 Bratislava, Slovakia; 3 SETI Institute, Mountain View, CA 94043, USA; 4 School of Medicine, Stanford University, Stanford, CA 94305, USA

**Keywords:** RNA–DNA differences (RDDs), reactive oxygen species (ROS), genomic instability, redox cycle, hypermutation, spaceflight

## Abstract

RNA–DNA differences (RDDs) challenge the traditional view of RNA as a faithful copy of DNA, arising through RNA editing, transcriptional errors, and oxidative damage. Reactive oxygen species (ROS) play a central role, inducing lesions like 8-oxo-guanine that compromise transcription and translation, leading to dysfunctional proteins. This review explores the biochemical basis of RDDs, their exacerbation under oxidative stress, and their dual roles in cellular adaptation and disease. RDDs contribute to genomic instability and are implicated in cancers, neurodegenerative disorders, and autoimmune diseases, while also driving phenotypic diversity. Drawing on terrestrial and spaceflight studies, we highlight the intersection of oxidative stress, RDD formation, and cellular dysfunction, proposing innovative mitigation approaches. Advancements in RDD detection and quantification, along with ROS management therapies, offer new avenues to restore cellular homeostasis and promote resilience. By positioning RDDs as a hallmark of genomic entropy, this review underscores the limits of biological adaptation. Furthermore, the prevalence of guanine-rich codons in antioxidant genes increases their susceptibility to ROS-induced oxidative lesions, linking redox stress, genomic instability, and constrained adaptation. These insights have profound implications for understanding aging, disease progression, and adaptive mechanisms in both terrestrial and space environments.

## 1. Introduction

### 1.1. RNA–DNA Differences: A Nexus Linking Oxidative Stress and Genomic Instability

The central dogma of molecular biology, defined as faithful RNA transcription from DNA templates, has been challenged by the discovery of RNA–DNA differences (RDDs). These deviations in RNA, including nucleotide substitutions, insertions, and deletions, fundamentally alter our understanding of gene expression and potentially disease mechanisms and therapeutic strategies. RDDs represent a significant departure from the genomic blueprint, demonstrating that in vivo RNA transcripts can diverge substantially from their DNA counterparts. This metabolically induced phenomenon is further complicated by the formation of G-quadruplexes (G4s), secondary structures found in the guanine-rich regions of both DNA and RNA, which can influence the transcript stability and [1,2,3]. These structures add another layer of complexity to the transcriptome—the complete set of RNA molecules (mRNA, rRNA, tRNA, and non-coding RNA) representing the functional readout of the genome [4].

RDDs arise through various mechanisms, including enzymatic editing, transcriptional errors, and oxidative damage [5]. Other contributing factors include template switching during transcription and chemical modifications of RNA bases, further expanding the potential sources of RDDs [6] While some RDDs may be viewed as being “normal biology”, resulting in proteomic diversity, others are considered mutations. In many cases, RDDs lead to stable alterations in protein sequences that are detectable via mass spectrometry. Importantly, mutations—particularly those induced by stressors like reactive oxygen species (ROS)—can disrupt cellular homeostasis. The stability of RDDs varies; some, like those from enzymatic editing, are reversible, while others persist, causing lasting functional consequences in cells. The high frequency of RDDs, with thousands of sites identified in various human tissues and cell types [7,8], underscores their potential impact on the cellular function.

Oxidative stress, caused by an overproduction of ROS within cells and tissues, may stem from disruptions in the electron transport chain (ETC). ROS, including superoxide anions (O_2_^−^), hydrogen peroxide (H_2_O_2_), and hydroxyl radicals (•OH), are highly reactive molecules. Endogenous metabolites like hydrogen sulfide (H_2_S) and carbon monoxide (CO) can exacerbate metabolic stress by inhibiting key ETC complexes, impairing electron flow and increasing ROS generation.

The ubiquitous presence of ROS, arising both metabolically and abiotically, drives genomic entropy, progressively degrading genomic integrity. This phenomenon indicates that mutation is not the exclusive domain of traditional evolutionary mechanisms. While Darwinian natural selection relies on mutations as a substrate for adaptation, oxidative stress primarily induces deleterious or neutral changes for the individual organisms that undergo RNA changes. Similarly, Lamarck’s emphasis on the inheritance of acquired characteristics, where organisms are thought to adapt to environmental pressures during their lifetime and pass these adaptations to their offspring [9], is limited under the persistent ROS burden. In such conditions, antioxidant defenses primarily buffer degradation rather than enable the kind of heritable, directed change that Lamarck envisioned.

Organisms with longer lifespans have more efficient antioxidant mechanisms to mitigate oxidative damage, while certain extremophiles have adapted to high-ROS environments through specialized strategies. For example, termite queens (*Reticulitermes speratus*) utilize large quantities of uric acid as an antioxidant, contributing to their extended lifespans [10]. Interestingly, humans and other higher primates also have higher levels of uric acid compared to other mammals due to the loss of a functional uricase enzyme [11]. This loss is hypothesized to be related to the antioxidant properties of uric acid. It scavenges ROS and contributes substantially to the antioxidant capacity of human plasma, although it also has potential drawbacks like an increased risk of gout [12]. Longer-lived bats exhibit higher antioxidant enzyme activities and more efficient DNA repair systems, which help compensate for their high metabolic rates and contribute to their longevity [13]. The extremophilic archaeon *Halobacterium salinarum* produces bacterioruberin, a carotenoid pigment that acts as an antioxidant, protecting against oxidative damage in high-salinity environments with intense UV radiation [14]. Radiation-resistant extremophiles like *Deinococcus radiodurans* possess efficient DNA repair systems, rapid removal of oxidative damage, and protective cellular mechanisms that mitigate the ROS effects [15].

Paradoxically, antioxidant genes (e.g., superoxide dismutase (SOD), thioredoxin, and glutathione synthase) are often encoded in guanine-rich regions with a bias toward guanine-rich codons, making them particularly vulnerable to ROS-induced lesions. Table 1 provides a summary of the genetic variations in antioxidant enzymes and their roles in neurological disease susceptibility. Table 1 specifically addresses neurological diseases due to their heightened vulnerability to oxidative stress and RDD-induced pathology.

In addition to the protective mechanisms provided by antioxidant genes, endogenous gaseous signaling molecules like hydrogen sulfide (H_2_S) and carbon monoxide (CO) play complex roles in the cellular redox balance. Elevated H_2_S can inhibit cytochrome c oxidase (Complex IV) in the ETC, disrupting electron flow and promoting ROS formation [23,24,25,26]. Similarly, CO binds to cytochrome c oxidase, inhibiting its activity and enhancing ROS production [27,28]. Interestingly, CO levels oscillate in conjunction with metabolic cycles, driven by the rhythmic expression of heme oxygenase 1 (HMX1/HO-1) [29,30]. These oscillations, potentially linked to the circadian clock, may influence metabolic rhythms and cellular function, with disruptions potentially contributing to metabolic and sleep disorders.

Beyond the roles of H_2_S and CO in regulating the redox balance, the rhythmic nature of cellular metabolism itself plays a crucial role in minimizing the oxidative damage to the genome. Cellular metabolism exhibits temporal compartmentalization, with DNA replication and a subset of RNA transcription occurring during the reductive phase of the metabolic cycle, characterized by lower ROS levels [31,32]. This minimizes the oxidative damage to the genome [31,33,34]. However, this segregation can be disrupted under stress conditions [35], with redox-sensitive signaling pathways dynamically influencing metabolic and transcriptional programs to maintain homeostasis [36]. The interplay between CO, H_2_S, and ROS oscillations is likely crucial for maintaining the redox balance, with potential synchronization or driving relationships to minimize the oxidative damage.

This review explores the intricate relationship between metabolic stress, redox biology, and genome instability, focusing on RDDs as a critical link between oxidative stress and genomic integrity. We examine the mechanisms by which metabolic stress drives RDD formation, their amplification under conditions of oxidative stress, and their functional consequences, including their impact on DNA repair, telomere maintenance, epigenetic regulation, and translation. We also discuss the potential of therapeutic interventions that address the underlying metabolic imbalances, such as the antioxidant, quercetin [37], and GlyNAC (glycine and N-acetylcysteine) [38], to mitigate RDD-associated pathologies. By elucidating the dual role of RDDs in both adaptation and disease, this review aims to provide insights into their potential as therapeutic targets for diseases rooted in genome instability and metabolic dysregulation.

### 1.2. Mechanisms of RNA–DNA Differences

RNA–DNA differences (RDDs) arise through two categories of processes: enzymatic RNA editing and non-canonical mechanisms. Canonical RNA editing, a phylogenetically conserved process, generates diverse protein isoforms and regulates gene expression by altering the nucleotide sequences within RNA. Two well-characterized forms are adenosine-to-inosine (A-to-I) and cytosine-to-uracil (C-to-U) conversions. A-to-I editing, mediated by ADAR enzymes, is prevalent in the brain, regulating the neuronal function and immune responses [39]. C-to-U editing, catalyzed by APOBEC enzymes, plays critical roles in lipid metabolism and immune signaling [40]. Additionally, the dynamic modification of N6-methyladenosine (m6A) in RNA is increasingly recognized for its roles in RNA splicing, stability, and translation, with dysregulation implicated in diseases like cancer.

Non-canonical RDDs encompass a broader range of nucleotide substitutions not directly attributable to known enzymatic processes. These often result from transcriptional errors, including misincorporation by RNA polymerase, RNA polymerase slippage (leading to insertions or deletions), template switching during transcription, or chemical modifications of RNA bases, particularly under oxidative stress. Among these, guanine oxidation to 8-oxo-7,8-dihydroguanine (8-oxoG) is the most significant and demonstrates the pervasive nature of oxidative damage. Tissues with a high metabolic activity or oxidative exposure, such as neural and cancerous tissues, exhibit heightened susceptibility to RDD formation through these mechanisms [41]. This lesion mispairs with adenine during transcription, causing G-to-A substitutions. It can also lead to G-to-T transversions during reverse transcription in vitro, impacting cDNA synthesis and downstream applications like PCR and sequencing. These effects are particularly pronounced in G-quadruplex regions in vivo, where oxidative damage impairs telomere maintenance and transcriptional regulation [42]. The combination of increased RDD formation in vulnerable tissues, the error-prone nature of specific lesions like 8-oxoG [43], and the impact on critical genomic regions like G-quadruplexes synergistically amplifies the mutagenic potential of RDDs, driving phenotypic changes and disease progression.

**Figure 1:**
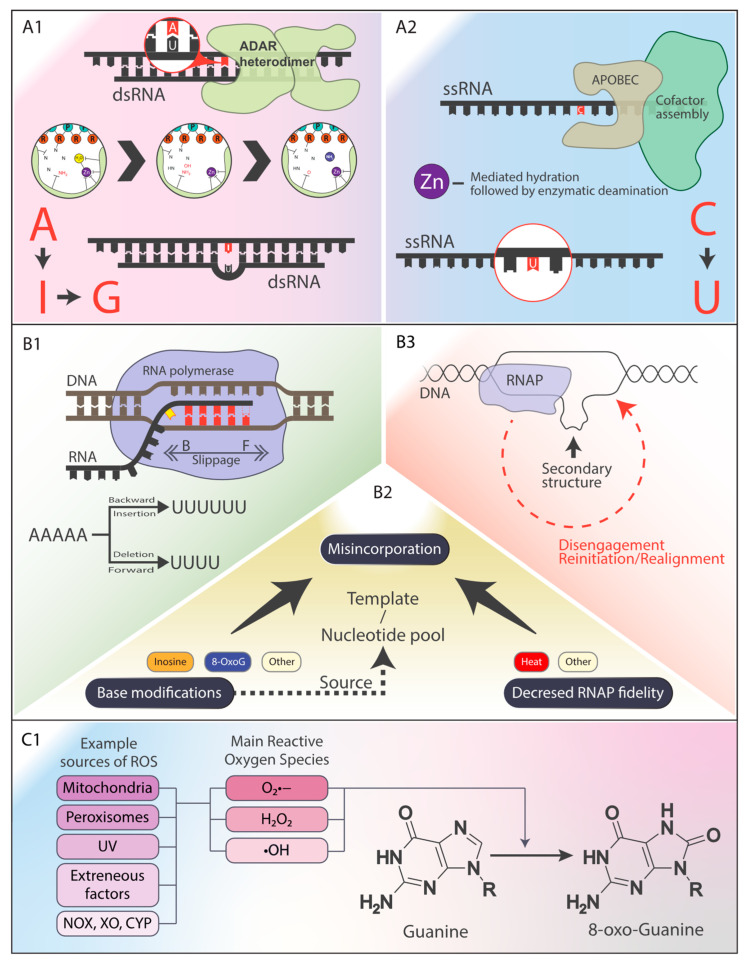
Mechanisms generating RNA–DNA differences (RDDs). RNA–DNA differences (RDDs) arise from diverse mechanisms that introduce sequence discrepancies between genomic DNA and transcribed RNA. This figure highlights key contributors to RDD formation: (**A**) enzymatic RNA editing: post-transcriptional modifications that alter RNA bases. (**A1**) ADAR enzymes: adenosine deaminases acting on RNA (ADARs) catalyze the hydrolytic deamination of adenosine (A) to inosine (I), which is interpreted as guanine (G) during translation, leading to A-to-G transitions. ADAR editing is critical for transcriptome diversity, particularly in repetitive elements like Alu sequences, and influences immune function and neural processes. Dysregulation is linked to diseases such as cancer and neurological disorders. (**A2**) APOBEC enzymes: while primarily involved in DNA editing and antiviral defense, certain apolipoprotein B mRNA editing catalytic polypeptide-like (APOBEC) enzymes can catalyze cytosine (C) deamination, often involving a zinc-coordinating active site for uracil (U) deamination in RNA. The specific functions and targets of APOBEC-mediated RNA editing remain under investigation. (**B**) Transcriptional errors: mistakes occurring during RNA synthesis. (**B1**) Polymerase slippage: RNA polymerase can slip at homopolymeric runs (e.g., AAAAA), leading to insertions or deletions (indels) in the transcript. This is more frequent in repetitive genomic regions. (**B2**) Misincorporation: incorrect nucleotide incorporation can arise due to the following: modified bases in DNA or the nucleotide pool (e.g., 8-oxoG mispairing); reduced polymerase fidelity caused by environmental stressors (e.g., high temperature) or mutations. Although proofreading and RNA surveillance mechanisms correct most errors, uncorrected misincorporation can contribute to RDD formation. (**B3**) Template switching: RNA polymerase may switch templates when encountering DNA secondary structures (e.g., hairpins, G-quadruplexes) or DNA lesions, producing chimeric RNA molecules. This mechanism is also exploited by some viruses (e.g., retroviruses) to enhance genetic diversity. (**C**) Oxidative damage: ROS-induced RNA modifications. (**C1**) 8-oxoG and ROS: 8-oxoguanine (8-oxoG) are major oxidative lesions generated by reactive oxygen species (ROS) arising from mitochondrial respiration, inflammation, and environmental stress (e.g., radiation, pollutants). During transcription, 8-oxoG mispairs with adenine (A), leading to G-to-T transversions in RNA. These oxidative modifications contribute to genomic and transcriptomic instability, potentially driving mutagenesis and disease. This figure provides a comprehensive overview of the mechanisms driving RNA–DNA differences, illustrating the dynamic nature of the transcriptome and its susceptibility to modifications and errors. Understanding RDDs is essential for elucidating gene regulation, protein diversity, and disease development.

Figure 1 provides a visual representation of the various mechanisms that contribute to RDD formation, which are described in detail in this section.

#### Oxidative Stress and the Formation of RDDs

Reactive oxygen species (ROS), including the superoxide anion, hydrogen peroxide, and hydroxyl radicals, have a dual nature. While essential for cellular functions like signaling and pathogen defense, ROS overproduction leads to oxidative stress, damaging the cellular components (DNA, lipids, and proteins). Generated during normal metabolism and exacerbated by environmental stressors, ROS are central to aging, disease development, and adaptation, influencing the cell signaling, apoptosis, immune defense, and stress responses in both plants and animals [44,45]. Cellular mechanisms regulate ROS levels, with antioxidant enzymes like superoxide dismutase (SOD) and catalase maintaining balance. SODs, crucial for antioxidant defense, catalyze superoxide anion (O_2_•−) dismutation into hydrogen peroxide (H_2_O_2_) and molecular oxygen (O_2_). Mammals have three main SOD isoforms, namely SOD1 (cytosolic), SOD2 (mitochondrial), and SOD3 (extracellular), each contributing to the redox balance and the protection of cells and tissues. The glutathione and thioredoxin systems also counteract oxidative stress using enzymes and small proteins to neutralize ROS. However, excessive ROS overwhelms these defenses, contributing to pathologies like cancer and neurodegenerative diseases, and even influences memory formation through oxidative stress, DNA damage, and epigenetic regulation [46]. In central nervous system disorders such as Alzheimer’s disease (AD), Parkinson’s disease (PD), stroke, amyotrophic lateral sclerosis (ALS), Huntington’s disease (HD), epilepsy, and migraine, oxidative stress often exceeds the brain’s antioxidative defenses [16].

In many cases, ROS are byproducts of normal metabolism, generated via mitochondrial respiration and NADPH oxidases, and are also induced by environmental factors like UV radiation and pollutants. In spaceflight, altered fluid dynamics due to microgravity, coupled with other stressors like radiation [47] and hypo-magnetic fields [48], can contribute to oxidative stress. ROS accumulate under stress (inflammation, radiation, microgravity, or mitochondrial dysfunction). Mitochondrial dysfunction, often linked to impaired electron transport chain (ETC) activity, is a major ROS source. The resulting oxidative stress increases RDD formation, compromising the cellular function and potentially contributing to genomic instability. RNA, due to its single-stranded nature and proximity to ROS-producing organelles like mitochondria and peroxisomes, is particularly susceptible to oxidative damage [49,50,51]. Guanine, with the lowest oxidation potential, is a primary target; its oxidation to 8-oxoG is a hallmark of oxidative stress [52]. This lesion mispairs with adenine during transcription, causing G-to-A substitutions in vivo, and can also lead to G-to-T transversions during reverse transcription in vitro, impacting cDNA synthesis and downstream applications like PCR and sequencing.

Importantly, while the generation of RNA–DNA differences (RDDs) can have biological consequences, as confirmed via mass spectrometry sequencing, the inherently transient nature of RNA significantly mitigates the potential negative impacts. Unlike the permanent genomic mutations in DNA, RNA molecules are rapidly turned over, degraded, and replaced through ongoing transcriptional activity. The cell employs robust RNA surveillance and quality control mechanisms—such as nonsense-mediated decay (NMD), no-go decay (NGD), and ribosome-associated quality control (RQC)—which effectively detect and eliminate faulty RNA transcripts, preventing harmful accumulation. Additionally, the high redundancy in mRNA copies ensures that even if errors arise in some RNA transcripts, numerous correctly transcribed mRNA copies remain functional, thereby greatly reducing the likelihood of significant adverse effects on the cellular physiology. Some RNA editing events or transcriptional errors produce silent mutations or synonymous codon substitutions, further minimizing their biological relevance due to a rapid protein turnover. By contrast, mutations within DNA carry greater severity and permanence, as they persist and propagate through subsequent cell divisions, potentially leading to heritable genomic instability and long-term functional disruption.

The presence of 8-oxoG in RNA has profound consequences for transcription and translation, leading to errors in RNA transcripts and protein synthesis. These effects can indirectly contribute to genomic instability by compromising the cellular processes involved in DNA maintenance. During transcription, it promotes mispairing with adenine, causing G-to-A transitions (detectable as RDDs). 8-oxoG can also disrupt the RNA structure, altering hairpin loops or affecting RNA–protein interactions, impacting RNA stability and function, such as interfering with RNA-binding proteins involved in splicing or translation regulation [53]. These effects are exacerbated by the fact that the repair mechanisms available for RNA are limited, compared to those for DNA. While DNA benefits from extensive and specialized repair pathways, such as base excision repair, nucleotide excision repair, and mismatch repair, to preserve its long-term stability as the cell’s primary genetic repository, RNA lacks comparable systems. Instead, damaged RNA is often degraded through mechanisms like nonsense-mediated decay (NMD) or no-go decay (NGD) and replaced with newly synthesized transcripts. This inherent limitation in RNA repair amplifies the vulnerability of RNA to damage, compounding the negative impacts on the cellular function and gene expression.

Cells employ antioxidant defenses (superoxide dismutase, catalase, thioredoxin, and glutathione peroxidase) to counteract ROS [54,55]. Hypometabolic states, with a reduced metabolic rate, offer a complementary strategy, limiting mitochondrial respiration (the primary ROS source) and thus oxidative damage to nucleic acids, reducing RDD formation. Beyond simply limiting ROS production, hypometabolism also upregulates endogenous antioxidant systems and DNA repair pathways, further enhancing the resistance to oxidative stress [56,57]. These adaptations, observed in various hypometabolic states such as hibernation, demonstrate the coordinated response to reduce oxidative damage [58,59]. This multifaceted approach to mitigating oxidative damage is strikingly apparent in long-lived hibernators such as the cancer-resistant naked mole-rat (*Heterocephalus glaber*), boasting a lifespan exceeding 37 years [60]. Similarly, hibernating bats (*Myotis brandtii*) can live for more than 40 years—approximately 10 times longer than expected for mammals of their size [61].

While these examples illustrate the benefits of minimizing ROS production, there are instances where ROS generation is a necessary and even beneficial process. Phagocytosis in human leukocytes exemplifies ROS-mediated RDDs. Upon immune activation, leukocytes generate a ROS burst to neutralize pathogens, but this can also cause oxidative modifications in nucleic acids. The resulting lesions, like 8-oxoG, can cause RDDs by impairing transcriptional fidelity. This highlights immune activation as a ROS source and the interplay between oxidative stress and transcriptome stability. This review focuses on the role of somatic genetic variations, particularly RDDs, in neutrophil function and their connection to ROS production and metabolic stress. While heritable genetic defects in neutrophils, such as those causing Chronic Granulomatous Disease (CGD), NCF4 variants, and p22^phox (CYBA) polymorphisms, are known to disrupt ROS production and immune responses [62,63], these represent distinct inherited conditions. Although genetic variations influencing neutrophils have been mapped in rats [64] and linked to ROS control [65], the current discussion specifically addresses acquired genetic changes (RDDs) arising from oxidative stress and metabolic dysfunction. These findings suggest that RDDs may play a broader role in health and disease by contributing to antibody diversity. While somatic hypermutation [66] is well established, this indicates additional diversification through RDDs in immunoglobulin genes. For example, NBH cells (a type of B-helper neutrophil) promote increased somatic hypermutation in marginal zone B cells, leading to higher mutation accumulation in the immunoglobulin VH3-23 gene, further diversifying the antibody repertoire [67]. While RDDs can contribute to beneficial diversity, it is essential that cells possess robust mechanisms to detect and repair oxidative DNA damage.

Both recent and older findings emphasize redox-mediated signaling in maintaining genome stability, crucial for the timely detection and repair of oxidative DNA damage, highlighting the interplay between oxidative stress and genome surveillance [68]. One critical mechanism involved in this process is DNA-mediated charge transport, used by base excision repair (BER) enzymes to detect base lesions.

BER enzymes like MutY and EndoIII, containing [4Fe4S] clusters, use DNA CT to scan the genome for oxidative damage. These repair proteins bind to DNA and can become oxidized to a [4Fe4S]^3+^ state, allowing them to transfer an electron through the DNA’s π-stack to another nearby repair protein in the [4Fe4S]^3+^ state, reducing it to [4Fe4S]^2+^ and causing it to dissociate. If a DNA lesion disrupts the π-stack, this charge transfer is blocked, and the oxidized protein remains bound near the lesion, marking it for repair.

The function of [4Fe4S] clusters, previously recognized primarily within base excision repair (BER) enzymes—which repair damaged DNA bases—has recently been expanded. Research now shows that the [4Fe4S] cluster within human DNA primase, the enzyme responsible for synthesizing RNA primers to initiate DNA replication, also functions as a redox switch, utilizing DNA charge transport (DNA CT) for rapid, long-range redox signaling [69,70]. This finding highlights the broader importance of these clusters in coordinating DNA replication and repair.

Oxidative stress can disrupt DNA-mediated charge transport, central to DNA replication and repair. Lesions like 8-oxoG and other oxidative lesions underscore the importance of cellular repair mechanisms like BER, with enzymes like 8-oxoguanine DNA glycosylase (OGG1) recognizing and excising 8-oxoG from DNA, preventing mutagenesis. However, excessive oxidative stress can overwhelm these repair systems, increasing the RDDs and genomic instability. Specifically, BER pathways, although essential for genome maintenance, introduce secondary damage that overwhelms the cellular repair machinery under chronic oxidative stress, highlighting the need for more precise therapeutic strategies targeting RDD reduction [6].

Oxidized guanine accumulation could signal to DNA primase—the enzyme that initiates DNA replication by synthesizing short RNA primers—that the cell has undergone enough metabolic cycles and is ready for DNA replication and cell division, ensuring cell division only when sufficient resources and energy are available [71]. The redox switch function of DNA primase could integrate metabolic signals with DNA replication and repair, adjusting its activity to match the cell’s metabolic status, thus ensuring the optimal conditions. It is important to note that primase requires access to the template DNA, which is enabled by upstream processes such as helicase-driven DNA unwinding, a process that indirectly relies on ATP [72,73].

Paradoxically, the redox sensing of DNA primase depends on the accumulation of oxidative damage in DNA—a consequence of adequate metabolic activity, which inevitably generates ROS as byproducts. This suggests a novel universal mechanism for timing cell division: cells could use the redox state of DNA, influenced by metabolically generated ROS, to signal the readiness for replication.

By dividing only when it has undergone enough metabolic cycles, based on the availability of essential nutrients, and when ready for DNA replication and cell division, the cell helps prevent population collapse. Such a collapse could occur if cells were prevented from completing their genome replication in a dynamically fluctuating environment that might suddenly become depleted of essential nutrients.

However, the elevated ROS levels associated with an increased metabolic activity also introduce a potential trade-off. While the redox state of DNA, influenced by these metabolically generated ROS, can signal the readiness for replication, the resulting oxidative damage can further compromise the genome stability. This damage contributes to the genomic instability and heterogeneity characteristic of cancer, highlighting the delicate balance between promoting cell division and maintaining the genomic integrity.

These findings provide insights into the cellular strategies for monitoring and maintaining the DNA integrity through redox-mediated signaling. Understanding these processes is vital for comprehending how disruptions in the redox balance can lead to genomic instability and disease.

**Figure 2:**
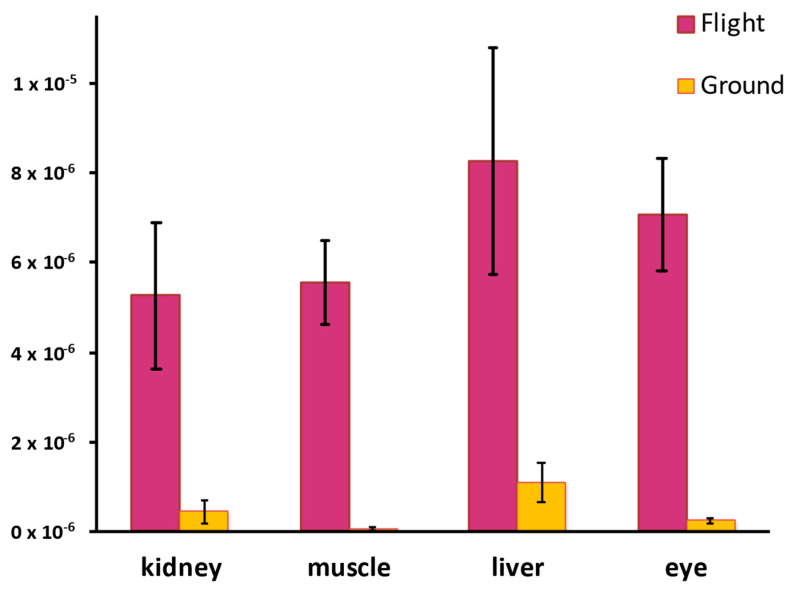
Tissue-specific RDD frequencies in spaceflight vs. terrestrial mice: evidence for oxidative stress-induced mutagenesis. Bar graphs represent mean RNA–DNA difference (RDD) frequencies measured across various tissues (kidney, muscle, liver, and eye) from mice exposed to 37 days of spaceflight (ISS) compared to terrestrial controls. Error bars indicate the standard deviation (SD) of replicate measurements (*n* ≥ 3 per group). Data shown are representative of repeated experiments to ensure reproducibility of observed patterns. Elevated RDD frequencies in spaceflight samples suggest increased oxidative stress-induced mutagenesis.

While cellular mechanisms maintain the redox balance and DNA integrity, environmental factors can disrupt these processes. Spaceflight, with combined stressors, challenges cellular homeostasis and increases the genomic instability risk. Research on ISS mice revealed that metabolic stress significantly drives RDD formation [5]. These mice displayed elevated ROS and increased RDD frequency, likely due to elevated CO_2_ and microgravity-induced metabolic changes, as shown in Figure 2. Spaceflight-induced RDDs were mapped to the ribosomal RNA gene *Rn45s*, with eye tissue showing significantly more unique SNVs as RDDs compared to liver and muscle tissue, highlighting the potential for ribosomal RNA mutations to disrupt protein synthesis and cellular function. This study links spaceflight-induced RDDs to oxidative damage driven by elevated ROS, exacerbated by metabolic stress and environmental factors, mirroring terrestrial conditions like hypoxia and diabetes, where disrupted cellular energy production leads to increased oxidative stress and RDDs [74]. This underscores metabolic stress as a universal RDD driver and the potential risks of prolonged exposure to such conditions, both in spaceflight and disease [75]. It remains to be determined whether these changes represent a potentially detrimental process or an adaptive stress response, particularly without direct evidence of functional impairment in this context.

The metabolic link to genetic instability has profound implications for various diseases. Elevated CO_2_ levels significantly contribute to ROS production and RDD formation. Carbon dioxide exacerbates oxidative stress by increasing hydroxyl radical (HO•) toxicity and promoting carbonate radical anion (CO_3_•^−^) formation from bicarbonate anion and hydroxyl radicals. The carbonate radical anion, a strong oxidant, can cause RDDs by oxidizing guanine.

While elevated CO_2_ is a spaceflight concern, it is also relevant to clinical scenarios like chronic obstructive pulmonary disease (COPD), where CO_2_ retention is common. In COPD, impaired lung gas exchange leads to elevated blood CO_2_ [76], which has been linked to increased oxidative stress [77,78]. This oxidative stress suggests a potential link to RDD formation, which may play a role in disease progression by impairing DNA repair, altering gene expression, and promoting inflammation. The link between elevated CO_2_, oxidative stress, and RDD formation observed in spaceflight underscores the broader relevance to terrestrial health. Studies in model organisms further illuminate this, demonstrating oxidative stress’s detrimental impact on the RNA integrity and cellular function. In yeast, hydrogen peroxide exposure increases RNA lesions, leading to translational errors and impaired growth [79]. In *C. elegans*, oxidative stress correlates with a reduced lifespan and reproductive capacity due to an increased RDD frequency [80].

In mammals, oxidative stress-induced RDDs contribute to various diseases. SOD1 knockout mice, lacking an essential antioxidant enzyme, exhibit heightened RNA oxidation, neurodegeneration, and motor deficits [81,82]. In humans, elevated oxidized RNA is found in neurodegenerative diseases like Alzheimer’s, where RDDs and disrupted protein synthesis, along with amyloid-beta plaque formation and tau protein hyperphosphorylation, appear to be interconnected with pathological features [83,84], suggesting a potential for complex bidirectional interactions. In cancer, the ROS-rich tumor microenvironment drives both genomic instability and RDD formation. RDDs in key transcripts, like those encoding the tumor suppressor, p53, disrupt the cellular regulation and enhance the tumor adaptability, highlighting oxidative stress’s dual role in promoting transcriptional and translational errors [85].

RDDs can contribute to cancer development by altering the expression of the genes involved in cell growth and regulation. For example, RDD-induced alterations in RNA transcripts represent a potential mechanism by which oncogene or tumor suppressor gene function could be impacted, although direct evidence explicitly linking specific RNA-level alterations to carcinogenesis requires further validation. This highlights the importance of understanding and mitigating RDD formation, particularly under chronic oxidative stress.

ROS play a complex role in cellular function. While essential for immune defense and signaling, excessive ROS can damage nucleic acids. Uncoupling protein 2 (Ucp2) exemplifies this duality. Ucp2 regulates ROS levels by modulating the mitochondrial membrane potential, reducing the electron leakage and ROS production. UCP2 gene knockout in mice increases ROS, enhancing the pathogen clearance but also causing tissue damage and immune dysfunction [86]. Interestingly, it also suppresses carcinogenesis [87], highlighting the complex interplay between ROS and cellular processes.

Some aspects of sepsis demonstrate the detrimental effects of excessive ROS. During sepsis, immune cells release large amounts of ROS, causing oxidative damage to a wide range of host tissues, including DNA damage and RNA damage. This can lead to increased RDDs and genomic instability, underscoring the pathological consequences of oxidative stress in immune overactivation [88,89].

## 2. Functional Consequences of RDDs

The consequences of RDDs are multifaceted, falling into three broad categories:

Protein dysfunction: By altering codons, RDDs can lead to incorrect amino acid incorporation during translation, producing misfolded or non-functional proteins. Confirmed via mass spectrometry sequencing, these changes represent bona fide mutations due to permanent amino acid sequence alterations. For instance, the oxidation-induced mutation of tryptophan codons (UGG) to stop codons (UAG) results in truncated proteins, often unstable and prone to aggregation [90]. These effects are particularly detrimental in neurodegenerative diseases, where protein aggregation contributes to synaptic dysfunction and cell death [91].

Genomic instability: Beyond protein dysfunction, RDDs can impact DNA repair pathways, telomere maintenance, and epigenetic modifications. RDDs could affect the expression or function of DNA repair enzymes, compromising DNA damage repair. Similarly, RDDs could influence the telomere length or the expression of telomere-associated proteins, potentially contributing to cellular senescence. Additionally, RDDs may alter the expression or activity of epigenetic modifiers, leading to changes in gene expression patterns. Furthermore, large-scale genomic instability can stem from oxidative damage, including chromosomal rearrangements arising from defects in repairing oxidative DNA damage (particularly abasic sites) or from nucleotide pool imbalances [92]. These imbalances can lead to errors in DNA replication and repair, further contributing to genomic instability. Therefore, large-scale genomic instability should be viewed as a potential consequence of oxidative stress and impaired DNA repair [93,94,95,96,97].

Immune responses: Disruption of G4 structures by RDDs can further exacerbate these consequences. G4s are crucial for the telomere integrity, and damage to telomeric G4s can lead to telomere shortening and genomic instability. Oxidative damage at telomeres, particularly the conversion of guanine to 8-oxoG, can disrupt the protective G-quadruplex structures, contributing to telomere shortening and genomic instability. This process is further exacerbated by the impaired excision of 8-oxoG by OGG1 due to the unique secondary structures at telomeres [6]. Additionally, G4s in gene promoters can regulate gene expression, and their disruption can lead to altered transcription and translation.

Table 2 summarizes the different RDD types and their potential consequences. By altering codons (mRNA nucleotide triplets specifying amino acids), RDDs can cause incorrect amino acid incorporation during translation, resulting in misfolded or non-functional proteins. These changes, confirmed via mass spectrometry sequencing, represent bona fide mutations due to amino acid sequence changes.

Beyond protein synthesis, RDDs can perform the following functions:

Inhibit RNase P activity: RDDs in tRNA can alter their structure and function, potentially inhibiting RNase P activity, a ribozyme essential for tRNA maturation. This inhibition can disrupt tRNA processing and protein synthesis, leading to cellular dysfunction [104,105]. Additionally, the catalytic RNA component of RNase P [106,107] can be oxidized, and the RDDs in this RNA can further inhibit its catalytic activity on pre-tRNAs.

Disrupt other RNP complexes: RDDs in the RNA components of various ribonucleoprotein (RNP) complexes can potently inhibit essential cellular processes. For example, RDDs in the signal recognition particle (SRP) RNA can impair its ability to target proteins to the endoplasmic reticulum (ER) for secretion, and ER stress is closely linked to ROS production as part of the unfolded protein response [108]. Similarly, RDDs in spliceosome RNA components can disrupt mRNA splicing, leading to aberrant protein production [109].

**Figure 3:**
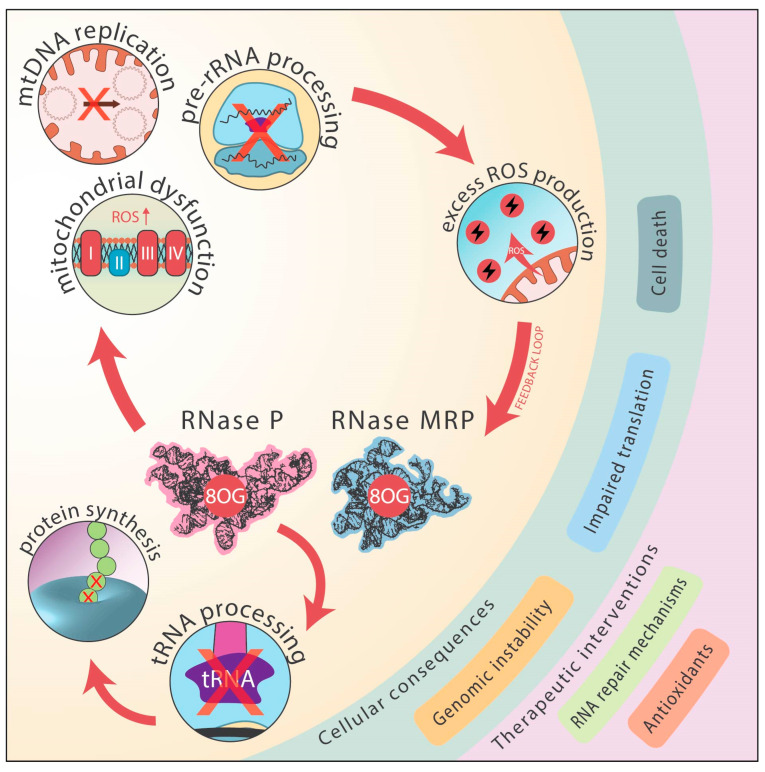
The vicious cycle of RNase MRP and RNase P RNA oxidation, mitochondrial dysfunction, and ROS amplification. This figure depicts the self-perpetuating cycle linking oxidative damage to RNase MRP and RNase P RNA with mitochondrial dysfunction and increased reactive oxygen species (ROS) production, ultimately driving further RNA oxidation and genomic instability.

RDDs in the RNA component of RNase P and MRP can impair its function in mitochondrial DNA replication and ribosomal RNA processing [104]. The impairment of RNase MRP function leads to mitochondrial dysfunction, a particularly critical issue because mitochondria are the primary site of ATP production, a process inherently coupled to the generation of ROS. There is no known alternative pathway for cells to produce ATP without the co-production of ROS. While certain processes (like glycolysis under fermentation) generate far less ROS than oxidative phosphorylation in mitochondrial ETCs, they are not entirely exempt from ROS formation. This inescapable biochemical reality creates a fundamental constraint. Mitochondrial dysfunction in eucaryotes, therefore, inevitably increases the ROS production, further exacerbating the RDD formation in RNase MRP and other RNA molecules, creating a potential vicious cycle. This feedback loop highlights a profound biochemical boundary. Figure 3 depicts the detrimental feedback loop between oxidative damage to RNase MRP and RNase P RNA, mitochondrial dysfunction, and ROS production. It demonstrates how the inherent need for efficient energy production via oxidative phosphorylation is inextricably linked to a continuous increase in genomic entropy, driven by ROS-induced damage. This entropic force, acting at the molecular level, has organismal consequences, potentially contributing to the observed variation in lifespan that correlates with the metabolic rate across different species. Organisms with higher metabolic rates tend to experience faster accumulation of ROS-induced damage, potentially leading to shorter lifespans, highlighting the trade-off imposed by this fundamental biochemical constraint. The discovery of RDDs supports the concept that organismal longevity often correlates inversely with the metabolic rate and the associated accumulation of oxidative damage and genome instability [110,111,112,113].

(1) Impaired RNA Function:

RNase MRP: Oxidative modifications to RNase MRP RNA compromise its crucial roles in mitochondrial DNA replication and pre-rRNA processing. This leads to impaired mitochondrial genome maintenance and ribosome biogenesis, reducing mitochondrial protein synthesis and ultimately causing defects in oxidative phosphorylation and diminished ATP production.

RNase P: Oxidative damage to RNase P RNA disrupts tRNA processing, resulting in an accumulation of improperly matured tRNAs and subsequent translation defects. This further exacerbates cellular stress and compromises the protein synthesis efficiency.

(2) Mitochondrial Dysfunction and ROS Amplification:

The combined effects of impaired RNase MRP and RNase P function lead to mitochondrial dysfunction, characterized by excessive ROS production. This heightened ROS environment further targets RNase MRP and RNase P RNA, amplifying oxidative RNA damage and establishing a vicious cycle.

The increased ROS levels drive the formation of additional RNA–DNA differences (RDDs), reinforcing mitochondrial instability and perpetuating a state of genomic and transcriptomic instability.

(3) Consequences and Broader Implications:

This feedback loop extends beyond mitochondrial dysfunction, as the continual accumulation of RDDs contributes to a decline in the overall genomic stability, impaired translation fidelity, and increased cellular vulnerability to oxidative stress.

Over time, this cycle accelerates the genomic entropy, underscoring the fundamental biochemical constraint that efficient energy production in the mitochondria is inherently linked to an inevitable increase in ROS-induced damage.

This interplay between oxidative stress and RNA modification highlights the fragility of cellular systems and the persistent challenge of maintaining the genomic and mitochondrial integrity in metabolically active environments. It also emphasizes the potential for therapeutic interventions targeting mitochondrial health and RNA quality control mechanisms to mitigate the detrimental effects of this vicious cycle.

Alter microRNA binding: RDDs in mRNA can affect the microRNA binding sites, leading to gene expression dysregulation and contributing to disease [114].

Change RNA localization: RDDs might influence RNA structure-based interactions required for trafficking and localization, affecting its functions [115].

Trigger immune response: RDDs can be recognized as “non-self” by cellular sensors, triggering innate immune responses and contributing to inflammation [116]. For example, RDDs can affect the Y-RNAs-based stress response, immune activation, and genomic stability [117].

Defective chromosome replication: Telomerase, a ribonucleoprotein enzyme complex, is crucial for maintaining telomere integrity [118]. Telomeres are protective caps at chromosome ends that safeguard genomic stability. The RNA subunit of telomerase, TERC, provides the template for telomere extension. However, TERC is highly susceptible to oxidative damage caused by reactive oxygen species (ROS), which impairs telomerase activity and accelerates telomere shortening. This, coupled with the inherent vulnerability of telomeric DNA to oxidative stress, promotes genomic instability and cellular senescence, a state of irreversible cell cycle arrest.

Crucially, the cellular redox state, particularly ROS levels, exhibits circadian rhythmicity and can influence the expression and activity of telomerase genes. This “redox switching” allows the circadian clock to integrate metabolic information and synchronize physiological processes, including telomere maintenance, accordingly. Disruptions in this intricate balance, through excessive ROS production or impaired redox regulation, can contribute to telomere dysfunction and accelerate the aging process [80].

Consequently, excessive ROS levels are closely linked to aging and age-related diseases. The oxidative damage inflicted upon both telomerase and telomeric DNA underscores the significant role of metabolic stress in driving cellular decline and genomic instability, ultimately contributing to the aging process.

In cancer, RDDs in oncogenes or tumor suppressor genes can promote cancer development and progression. For example, RDDs in the p53 transcript can disrupt its function, leading to uncontrolled cell growth and tumor formation. In aging, oxidative stress and RDD accumulation contribute to cellular senescence and proteome integrity decline. Age-related diseases often feature elevated RNA oxidation, linking chronic RDD accumulation to progressive cellular dysfunction [119,120].

### 2.1. Cellular Mechanisms to Counteract RDDs

The cellular complement of phylogenetically invariant and overlapping biochemical mechanisms mitigating RNA oxidation (which creates genome instability) testifies to the damaging omnipresence of ROS generated by the metabolic process in the ETC. Table 3 is relevant to the discussion of how cells counteract RDDs.

Nonsense-mediated decay (NMD): NMD is a critical mRNA surveillance mechanism that identifies and degrades transcripts containing premature termination codons (PTCs), which can arise from mutations or RDDs. By eliminating these faulty mRNAs, NMD prevents truncated, potentially harmful protein synthesis. The process involves PTC recognition, SURF complex (SMG-1, UPF1, eRF1, and eRF3) assembly, and the recruitment of RNA degradation machinery [126].

No-go decay (NGD): NGD addresses ribosomal stalling during translation, which can result from obstacles like strong RNA secondary structures or oxidative lesions. When a ribosome stalls, NGD detects it and initiates endonucleolytic cleavage near the stall site, followed by mRNA fragment degradation by exonucleases [127].

Ribosome-associated quality control (RQC): RQC manages incomplete nascent peptide degradation resulting from stalled translation. Upon ribosomal stalling, RQC facilitates ribosomal subunit dissociation and targets the incomplete polypeptide for ubiquitination and proteasomal degradation, preventing defective protein accumulation [127].

**Figure 4:**
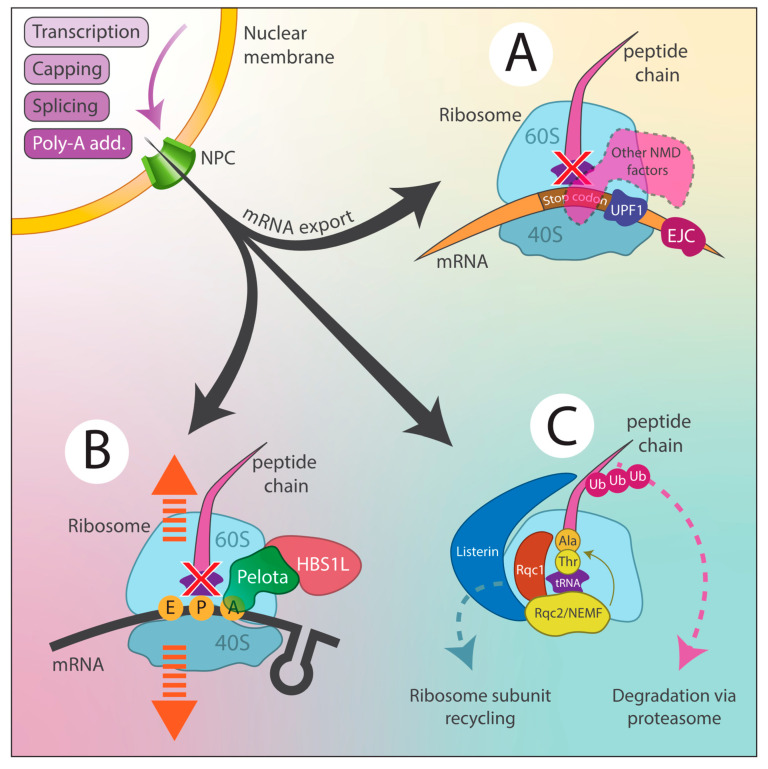
Cellular mechanisms for counteracting RDDs. This figure illustrates the key quality control pathways that recognize and degrade faulty mRNAs or proteins arising from RNA–DNA differences (RDDs), ensuring proper cellular function. (**A**) Nonsense-mediated decay (NMD). A mRNA surveillance pathway that prevents the accumulation of truncated, potentially toxic proteins. UPF1, a central RNA helicase, recognizes premature stop codons, triggering a cascade of events: decapping (removal of the 5′ cap). Deadenylation (removal of the 3′ poly(A) tail). Exonucleolytic degradation by the exosome and XRN1, ensuring that faulty transcripts are eliminated. (**B**) No-go decay (NGD). A response to ribosome stalling caused by structural obstacles, damaged codons, or defective RNA. Dom34 (Pelota) and Hbs1 recognize the stalled ribosome and cleave the problematic mRNA near the stall site. The truncated mRNA fragments are degraded by exonucleases such as XRN1 and the exosome complex. The stalled ribosome is recycled, preventing translation bottlenecks. (**C**) Ribosome-associated quality control (RQC). Ensures degradation of incomplete polypeptides that stall on ribosomes. Listerin (LTN1), an E3 ubiquitin ligase, ubiquitinates the stalled polypeptide, marking it for proteasomal degradation. NEMF (Rqc2 homolog) facilitates CAT tail (C-terminal alanine–threonine extension) addition, signaling faulty peptides for disposal. ANKZF1 (Vms1 homolog, not displayed) cleaves stalled peptides and aids in ribosome recycling.

These quality control pathways are essential for maintaining cellular homeostasis by ensuring gene expression fidelity. However, during chronic oxidative stress, increased oxidative lesions can overwhelm these systems, leading to aberrant mRNA and defective protein accumulation, contributing to cellular dysfunction and disease. The efficiency of these pathways can be influenced by the oxidative stress severity, cell type, and specific genes affected. In addition to these core pathways, RNA helicases help resolve the RNA secondary structures induced by oxidative lesions, while exoribonucleases degrade rather than repair aberrant RNAs. Cells also activate the stress response pathways, such as the unfolded protein response, to cope with misfolded proteins caused by RDDs. Figure 4 illustrates the three main quality control pathways (NMD, NGD, and RQC) that cells use to mitigate the effects of RDDs.

RDD identification and characterization often rely on high-throughput sequencing [5] and proteomics [128].

### 2.2. The Clinical Potential of RNA–DNA Differences: Neoantigens in Cancer Immunotherapy and Autoantigens in Autoimmune Diseases

RDDs offer a novel layer of transcriptome complexity with implications for oncology and immunology. These discrepancies between RNA transcripts and DNA templates can result from RNA editing, transcriptional errors, or environmental factors like oxidative stress. Table 4 provides an overview of RDD clinical implications. In cancer immunotherapy, RDDs can generate unique neoantigens, tumor-specific epitopes absent from normal tissues [129]. Conversely, in autoimmune diseases like systemic lupus erythematosus (SLE), RDDs may contribute to autoantigen generation, triggering inappropriate immune responses [117]. This dual role highlights both the therapeutic opportunities and challenges posed by RDDs in clinical medicine. However, potential challenges in developing RDD-based therapies, such as off-target effects or disrupting normal immune regulation, must be considered.

### 2.3. RDDs as Neoantigens in Cancer Immunotherapy

Neoantigens are tumor-specific antigens arising from genetic or transcriptomic alterations within cancer cells. Highly immunogenic, they are absent in normal tissues and thus evade central immune tolerance. RDDs, particularly those from RNA editing or oxidative stress, expand the potential neoantigen repertoire by introducing RNA-level variations distinct from the DNA sequence.

RNA editing, particularly adenosine-to-inosine (A-to-I) editing mediated by ADAR enzymes, plays a prominent role in generating RDDs. This process alters mRNA codons, leading to novel, potentially immunogenic peptides [137]. Many cancers exhibit dysregulated RNA editing, increasing the likelihood of unique neoantigen generation. For instance, the A-to-G transition (a hallmark of ADAR-mediated editing) has been observed to create novel amino acid sequences that can bind to major histocompatibility complex (MHC) molecules, enhancing T-cell recognition. Specific examples include RDD-derived neoantigens identified in melanoma and lung cancer that elicit strong T-cell responses [138].

In addition to RNA editing, oxidative stress within the tumor microenvironment contributes to RDD formation. Tumors often exhibit elevated ROS levels, which can oxidize guanine to 8-oxo-7,8-dihydroguanine (8-oxoG). This oxidative lesion introduces G-to-A transitions during transcription, creating RNA-level alterations differing from the corresponding DNA. These RDDs may produce tumor-specific peptides, serving as highly immunogenic neoantigens because they are absent from the thymic repertoire and less likely to induce tolerance.

The clinical application of RDD-derived neoantigens in cancer therapy is promising. Integrating RNA sequencing data allows the identification of neoantigens arising from RDDs, complementing DNA-based predictions. Computational tools can predict neoantigen binding affinity to MHC molecules, aiding the selection of the most immunogenic candidates. These neoantigens could be incorporated into peptide-based vaccines or used to engineer T-cell receptors for adoptive cell therapies, CAR T-cell therapy, and mRNA-based cancer vaccines. Tumors with high ROS levels or dysregulated RNA-editing enzymes (e.g., glioblastomas or hepatocellular carcinomas) are ideal candidates for exploring RDD-derived neoantigens. Gifford and colleagues have pioneered methodologies using machine learning and advanced bioinformatics to enhance the neoantigen design [139]. For example, PUFFIN (Predictor of Uncertainty in Peptide-MHC Interactions), a deep residual network model, predicts peptide–MHC binding affinities and quantifies the prediction uncertainty. By incorporating both structural features and binding affinities, PUFFIN enables the more precise identification of potential immunogenic epitopes.

Neoantigens arising from RDDs hold immense promise for revolutionizing cancer immunotherapy, offering a personalized approach by targeting aberrant peptides generated through transcriptional and post-transcriptional alterations. Peptide-based vaccines targeting RDD-derived neoantigens can elicit robust T-cell responses, training the immune system to recognize and attack cancer cells with enhanced specificity and efficacy [140]. RDD-derived neoantigens are also ideal targets for engineering T-cell receptors (TCRs) in adoptive cell therapies, enabling precise tumor cell elimination [141]. This extends to chimeric antigen receptor (CAR) T-cell therapy, broadening its application beyond surface antigens to target intracellular neoantigens from RDDs [55]. Encoding RDD-derived neoantigens in mRNA vaccines can direct a targeted anti-tumor response, particularly promising for cancers with high mutational burdens or unique transcriptomic profiles [142]. Integrating RDD-derived neoantigens into immunotherapy offers a powerful new paradigm for personalized cancer treatment, leveraging these unique molecular signatures to develop precision therapies and drive the future of cancer immunology.

Integrating RNA sequencing data with these computational tools expands the pool of potential RDD-derived neoantigens, especially in cancers with high ROS-induced RDDs or dysregulated RNA editing, enhancing the immunotherapy precision and efficacy by targeting a broader spectrum of tumor-specific alterations, including those from the dynamic tumor microenvironment. However, tumor heterogeneity and the potential for RDDs to vary within tumor cell subpopulations must be considered, as this could impact the RDD-based immunotherapy design and efficacy.

#### RDDs as Autoantigens in Autoimmune Diseases

While RDDs enhance the immune recognition in cancer, they may also contribute to the breakdown of the immune tolerance in autoimmune diseases. Autoimmune disorders (e.g., systemic lupus erythematosus (SLE) and rheumatoid arthritis (RA)) are characterized by autoantibody generation against self-antigens. RDDs, particularly those from aberrant RNA editing or oxidative stress, can generate modified peptides perceived as foreign, promoting autoimmunity.

RNA editing abnormalities are common in autoimmune diseases. Dysregulated ADAR activity, for example, has been observed in SLE. Aberrant editing introduces RDDs that may result in modified peptides recognized as non-self. For instance, RDDs in Ro and La ribonucleoproteins (common SLE autoantigens) may alter their structure and enhance immunogenicity [117]. These peptides, presented by MHC molecules, can activate autoreactive T-cells and drive autoantibody production, contributing to autoimmune disease pathogenesis. Oxidative stress, a hallmark of many autoimmune diseases, further amplifies this process, with elevated ROS levels leading to RNA oxidation and generating additional RDDs that can act as autoantigens.

RDDs can alter RNA structures, enhancing their recognition by innate immune sensors such as Toll-like receptors (TLRs). Specifically, single-stranded RNAs (ssRNAs) with certain modifications can activate TLR7 and TLR8, leading to pro-inflammatory cytokine production and perpetuating inflammatory cycles. This mechanism contributes to the innate immune dysregulation in autoimmune diseases [116,143,144].

Clinically, RDDs present both diagnostic and therapeutic opportunities in autoimmunity. Profiling the RNA editing levels or identifying specific RDD signatures could serve as biomarkers for disease diagnosis or progression. For example, specific RDD patterns in peripheral blood cells could be used for early diagnosis or to monitor the disease activity in SLE patients. Therapeutically, modulating RNA editing pathways or reducing oxidative stress may help mitigate the autoantigenic potential of RDDs. Specific strategies include RNA editing inhibitors, antioxidants, or targeted therapies that modulate immune responses to RDD-derived autoantigens. However, such interventions must be carefully tailored to avoid disrupting the normal immune regulation.

## 3. Balancing Therapeutic Potential and Pathogenic Risks

The dual role of RDDs—as beneficial neoantigens in cancer and harmful autoantigens in autoimmunity—highlights their complexity in clinical applications. Leveraging RDDs therapeutically requires a nuanced approach that maximizes their immunogenic potential in oncology while minimizing their contribution to autoimmunity. Future research should elucidate the mechanisms by which RDDs influence immune recognition in different contexts and develop precision therapies targeting these processes. This includes personalized approaches considering individual RDD profiles and the immune status to maximize the therapeutic benefits and minimize the risks.

Integrating RDD analyses into cancer immunotherapy pipelines and autoimmune disease studies could yield transformative insights. High-throughput sequencing, combined with advanced computational tools, is essential for mapping RDDs with single-nucleotide resolution and understanding their immunological impact. By unraveling the interplay between RDDs, immune recognition, and disease, innovative therapies harnessing their unique potential may be developed. However, the ethical considerations associated with manipulating RDDs therapeutically, such as potential off-target effects and the need for careful monitoring, must be addressed.

### 3.1. Adaptive and Clinical Implications

While potentially disruptive, RDDs may confer adaptive advantages by enhancing the transcriptomic diversity, which could be beneficial under fluctuating environmental conditions, enabling populations to survive stress and adapt to novel challenges. For instance, RDDs in immune cells could contribute to antibody diversity, facilitating broader pathogen recognition. Similarly, RDDs in stress response genes could promote the adaptation to changing environments (e.g., temperature fluctuations or nutrient availability). This inherent flexibility may have contributed to the resilience and adaptability observed across diverse species.

Clinically, RDDs have significant diagnostic and therapeutic potential. Sepsis tissue may contain the maximum number of RDDs. Sepsis, characterized by a systemic inflammatory response to infection, leads to widespread tissue damage and organ dysfunction. During sepsis, immune cells release large amounts of ROS to kill pathogens; however, excessive ROS can also damage host cells and tissues, including DNA and RNA, leading to increased RDD frequency. Other factors contributing to increased RDDs in sepsis tissue include inflammation (which can activate RDD-causing enzymes), hypoxia (which can increase ROS production and RDD formation), and mitochondrial dysfunction (which can also increase ROS production and RDD formation). The increased RDD frequency in sepsis tissue may contribute to sepsis pathogenesis by altering protein function and disrupting cellular processes. RDDs may also serve as biomarkers for sepsis diagnosis and prognosis.

Our recent findings have significant implications for human health in both spaceflight and terrestrial settings. In spaceflight, the combined effects of microgravity and elevated carbon dioxide can increase oxidative stress and RDD formation, potentially affecting astronaut health during long-duration missions [5]. Mice exposed to the space environment exhibit increased RDD frequency, likely due to these combined effects [145]. These RDDs can lead to non-synonymous mutations (NSMs), potentially altering protein function and disrupting essential cellular processes. On the Earth, RDDs may play a role in the conditions associated with oxidative stress, such as hypoxia, ischemia-reperfusion injury, and metabolic diseases like diabetes, where disruptions in cellular energy production and utilization lead to increased oxidative stress [146,147,148]. The potential risks associated with increased RDD formation due to metabolic stress and elevated ROS in space are a significant concern for astronaut health, especially during long-duration missions.

The ability to identify and characterize RDDs, including those resulting in altered protein sequences (confirmed via mass spectrometry), paves the way for a deeper understanding of disease mechanisms and the targeted interventions. As biomarkers, RDDs could signal the presence of various diseases. For example, distinct RDD patterns in peripheral blood cells may enable early diagnosis or monitor the disease activity in autoimmune conditions like SLE. In cancer, RDD profiles could offer insights into tumor subtype, prognosis, and potential therapy responsiveness. Moreover, RDD analysis could facilitate patient stratification based on the disease subtype or risk, enabling more personalized treatment strategies. In cancer, identifying specific RDDs could predict the responses to immunotherapy or guide targeted therapy selection. Furthermore, tracking changes in RDD profiles over time could yield valuable information about the disease progression and treatment response, particularly in chronic conditions like neurodegenerative or autoimmune diseases [149,150].

Therapeutically, strategies could be devised to modulate RDD-generating mechanisms. In cancer, inhibiting RNA-editing enzymes or mitigating oxidative stress could limit neoantigen formation and potentially augment the immunotherapy efficacy. While the potential of these therapeutic strategies is promising, they are largely theoretical and require further research to establish their clinical efficacy. In contrast, GlyNAC (glycine and N-acetylcysteine) has demonstrated effectiveness in mitigating metabolic stress and improving the redox balance in a randomized clinical trial, providing more robust evidence for its therapeutic application against excess ROS [38].

The randomized clinical trial investigating GlyNAC underscores its potential for mitigating the metabolic stress caused by excess ROS, which drive oxidative damage across multiple biological systems. By significantly enhancing the intracellular glutathione levels, GlyNAC restores the redox balance, reducing oxidative stress and improving the mitochondrial function—key contributors to cellular and genomic integrity. Excess ROS not only damage DNA but also affect RNA, increasing the RDD frequency. These RDDs arise from oxidative modifications of nucleotides, such as 8-oxoG formation, which can interfere with the transcriptional fidelity. By reducing the ROS levels, GlyNAC supplementation has the potential to minimize RNA oxidative modifications, thereby lowering RDD occurrence and preserving transcriptomic integrity, essential for accurate protein synthesis and cellular function. The trial further demonstrated systemic benefits, such as reduced inflammation, improved insulin sensitivity, and enhanced muscle performance. These outcomes suggest that ROS-targeting interventions like GlyNAC may have broader implications for maintaining the genomic and transcriptomic stability, particularly under the conditions associated with high oxidative stress, such as aging, metabolic disorders, and mitochondrial dysfunction. By mitigating ROS-induced damage, GlyNAC holds promise not only for improving the metabolic health but also for reducing the genomic instability driven by oxidative stress, including RDD suppression. This dual action highlights its potential as a therapeutic strategy for maintaining both metabolic and genomic integrity.

### 3.2. Biochemical Explanation for GlyNAC’s Effectiveness in Reducing ROS

GlyNAC’s ability to reduce ROS stems from its dual action on the glutathione and hydrogen sulfide (H_2_S) pathways [151]. N-acetyl cysteine (NAC), a widely used antioxidant in clinical trials, animal studies, and cell culture experiments, is a cornerstone of oxidative stress research and is also marketed as a dietary supplement. NAC is metabolized to cysteine, which fuels both glutathione synthesis and H_2_S production. Glycine contributes to glutathione synthesis by providing the glycine moiety. H_2_S is oxidized in the mitochondria to form sulfane sulfur species (persulfides and polysulfides), potent ROS scavengers that neutralize oxidative molecules and protect cellular macromolecules. This multifaceted action effectively combats oxidative stress, provided that H_2_S levels remain physiological.

The biochemical synergy between glycine, NAC, and their metabolites explains GlyNAC’s broad cytoprotective effects. Elevated ROS levels are implicated in various pathologies, including aging-related disorders, cancer, and neurodegenerative diseases. GlyNAC counteracts this by boosting the glutathione levels and promoting sulfane sulfur species generation, directly targeting the ROS cascade. However, careful H_2_S concentration control is needed due to its dual role in ROS modulation. GlyNAC’s balanced formulation is crucial for maintaining optimal H_2_S levels and maximizing the therapeutic benefits. This mechanism is particularly important as ROS-induced damage can lead to RDDs and genomic instability. GlyNAC’s dual action provides robust protection against these disruptions, improving the genomic stability and cellular resilience.

## 4. Therapeutic Insights

GlyNAC’s synergistic action highlights its therapeutic potential as a ROS-modulating agent. By simultaneously supporting glutathione synthesis and promoting the H_2_S-mediated antioxidative pathways, it addresses oxidative stress through multiple complementary mechanisms, crucial for conditions driven by redox imbalances. Maintaining the optimal H_2_S concentrations is essential due to its dual role as both a protective agent and a potential stressor. GlyNAC’s balanced composition appears to achieve this equilibrium effectively, making it a potentially superior therapeutic intervention compared to standalone antioxidants.

GlyNAC’s success in clinical trials targeting oxidative stress can be attributed to its biochemical synergy, enhancing both glutathione synthesis and H_2_S production. These pathways converge to produce sulfane sulfur species, which directly neutralize ROS and restore the redox balance. These mechanisms position GlyNAC as a promising therapeutic agent for managing oxidative stress-related conditions. Future research should explore optimized formulations and dosing strategies to maximize the clinical benefits while mitigating the potential risks associated with excessive H_2_S production.

In addition to targeted therapies like GlyNAC, ensuring adequate levels of essential vitamins is crucial for supporting the immune function, mitigating oxidative stress, and promoting the recovery from sepsis. Vitamin C, a potent antioxidant, has shown promise in reducing inflammation and organ dysfunction in sepsis patients [152]. Vitamin D plays a critical role in immune regulation, and its deficiency has been linked to worse sepsis outcomes. Studies indicate that vitamin D deficiency is prevalent among ICU patients, and supplementation may support the immune function in this context [153]. Thiamine (vitamin B1) is essential for cellular energy production and is often used with vitamin C in sepsis treatment. Some studies suggest that thiamine supplementation may improve lactate clearance and reduce the vasopressor requirements in septic patients, although its impact on survival remains uncertain [154]. Vitamin E, another powerful antioxidant, may also help protect against ROS-induced damage. By supporting these vital functions, adequate vitamin levels may indirectly contribute to reducing RDD formation and promoting genomic stability during sepsis recovery. However, further research is needed to establish the direct impact of vitamin supplementation on RDD formation in sepsis patients.

In autoimmune diseases, modulating RNA editing pathways or reducing oxidative stress may help curb the autoantigenic potential of RDDs. Another approach could involve bolstering cellular mechanisms that counteract RDDs, such as nonsense-mediated decay (NMD), no-go decay (NGD), and ribosome-associated quality control (RQC), to maintain cellular homeostasis and prevent aberrant protein accumulation. This could be achieved through drugs that enhance these pathways’ activity or by employing gene therapy. Furthermore, developing RNA repair mechanisms based on DNA repair pathways or using CRISPR-based approaches to correct RDDs could offer a more direct means of addressing the RDD consequences [155,156]. However, this remains a nascent field requiring further investigation into the feasibility and safety of such approaches.

Personalized medicine could also leverage RDD profiles to guide treatment decisions. In cancer, identifying specific RDD-derived neoantigens could inform personalized vaccine or adoptive cell therapy design. In autoimmune diseases, understanding an individual’s RDD profile could help tailor immunosuppressive therapies or identify patients who might benefit from targeted interventions.

Despite the immense clinical potential of RDDs, several challenges persist. Accurately identifying and quantifying RDDs necessitates sophisticated high-throughput RNA sequencing and robust bioinformatics tools. Further advancements in these areas are crucial for enhancing the RDD detection sensitivity and specificity. Moreover, the complexity of RDDs, influenced by the genetic background, environmental exposures, and disease state, must be fully understood to develop effective diagnostic and therapeutic strategies. Finally, manipulating RDDs therapeutically raises ethical considerations, such as potential off-target effects and the need for careful monitoring. Establishing clear guidelines and ethical frameworks for RDD-based therapies is essential.

Despite these challenges, the future of RDD research is promising. Continued refinement of antioxidant therapies and redox balancing strategies will benefit from the foundational chemical insights into free radical behavior established in early redox studies [157]. As our understanding of RDDs deepens and technologies advance, we can anticipate a growing number of clinical applications harnessing these transcriptomic variations. RDD-based diagnostics and therapies hold the promise of revolutionizing personalized medicine and improving the patient outcomes across a wide range of diseases [158].

Clinically, the role of RDDs in disease highlights the opportunities for therapeutic intervention. Antioxidant therapies (e.g., N-acetylcysteine, trimethylglycine, vitamin E, coenzyme Q10, and quercetin) and targeted antioxidant enzymes could reduce oxidative stress and thereby minimize RDD formation. Enhancing the RNA surveillance mechanisms or developing enzymatic tools to repair RNA lesions (based on DNA repair pathways or using CRISPR-based approaches) may further mitigate the RDD effects.

### Hypometabolism as a Therapeutic Intervention

Regulated hypometabolism offers a significant clinical potential beyond its adaptive role in nature, particularly for managing the conditions linked to oxidative stress and promoting health in extreme environments. Studies on hypometabolic animals exposed to radiation have revealed numerous protective effects. These include enhanced survival rates, demonstrating that hypometabolism appears to confer a survival advantage in the face of radiation exposure. Additionally, apoptosis and necrosis are significantly reduced in various cell types, such as blood lymphocytes, bone marrow hematopoietic cells, and thymus cells [56,57]. Furthermore, hypometabolism helps maintain the structural integrity of cells, mitigating radiation-induced damage [58]. These findings suggest promising applications for hypometabolism in various contexts, including space travel and clinical medicine.

In space travel, astronauts face elevated radiation levels and microgravity, both of which contribute to genomic instability and RDD formation. Inducing hypometabolism could offer a protective strategy against these spaceflight-related stressors [159,160]. In clinical settings, hypometabolism may prove beneficial in managing conditions characterized by acute or chronic oxidative stress, such as ischemia-reperfusion injury, neurodegenerative diseases, and aging [161].

While the therapeutic potential of hypometabolism is substantial, realizing its full clinical application requires further research. This includes identifying reliable ways to induce and maintain hypometabolism in humans without adverse effects and thoroughly investigating the long-term impacts of hypometabolism on the genomic and transcriptomic stability. Synthetic torpor represents a transformative approach for managing oxidative stress during long-duration spaceflight and clinical ischemia-reperfusion injuries [162,163]. For a comprehensive overview of therapeutic strategies targeting RDDs, refer to Table 5.

Mitigation Strategies for RNA–DNA Differences (RDDs)

Mitigating RDD formation and its associated impacts involves a multifaceted approach targeting oxidative stress reduction, enhanced cellular repair mechanisms, and novel therapeutic interventions. The key strategies include the following:

1. Oxidative stress reduction: Antioxidant therapies such as N-acetylcysteine (NAC), GlyNAC, quercetin, and vitamin E can enhance cellular defenses against ROS. Modulating the gasotransmitter levels (CO and H_2_S) offers additional control by preventing excess ROS production linked to mitochondrial dysfunction.
2. Enhanced RNA and DNA repair: Strengthening cellular pathways like nonsense-mediated decay (NMD), no-go decay (NGD), and ribosome-associated quality control (RQC) ensures the more efficient degradation of aberrant RNA molecules. Emerging RNA repair technologies modeled on DNA repair pathways, such as CRISPR-Cas13-based editing, present promising tools for directly correcting RDDs.
3. Advanced detection and quantification: High-throughput sequencing and mass spectrometry, coupled with bioinformatics tools like PUFFIN, allow for the precise mapping and monitoring of RDDs, providing insights into their formation and functional consequences.
4. Therapeutic modulation of RNA editing: Targeted modulation of ADAR and APOBEC enzymes can either inhibit harmful RNA editing in cancer or enhance the beneficial editing in other contexts, offering potential therapeutic leverage.
5. Disease-specific approaches: In cancer, RDD-derived neoantigens present opportunities for personalized immunotherapies, including mRNA vaccines and CAR T-cell therapies. For autoimmune diseases, reducing oxidative stress and modulating RNA editing pathways could mitigate autoantigenic RDDs.
6. Personalized medicine and spaceflight applications: Personalized RDD profiles can inform tailored therapies, while spaceflight-specific interventions, such as environmental controls and radiation shielding, address unique oxidative stress challenges.

## 5. Conclusions

RNA–DNA differences (RDDs) represent a critical dimension of transcriptomic complexity, with far-reaching implications for cellular function and disease. They are not merely passive variations but can represent stable, heritable changes in the genetic code—bona fide mutations—capable of altering the protein function and cellular behavior. However, not all RDDs are equivalent; some, particularly those from RNA editing, may be reversible and contribute to normal cellular processes. Oxidative stress is a major driver of RDD formation, linking environmental and metabolic stressors to transcriptomic and genomic instability. Understanding the mechanisms underlying RDDs and their impact on health and disease is pivotal for developing strategies to restore cellular homeostasis and improve the therapeutic outcomes. Future research should focus on developing more refined technologies to identify and quantify RDDs and creating targeted therapies to modulate RDD formation or mitigate their effects. Moreover, advancing technologies that effectively manage ROS and RDDs could not only reduce transcriptomic and genomic instability but also play a critical role in extending the lifespan by preserving the cellular function and delaying the onset of age-related diseases.

Importantly, the ongoing phylogenetic analysis of genomes (including extremophiles) for both ROS-generating and -detoxifying enzymes, correlating this with lifespan, including studying human centenarians for low-frequency SNPs linked with ROS management [165,166], represents an important data mining strategy. This approach could reveal novel insights into the genetic and molecular mechanisms underlying longevity and resilience to oxidative stress, potentially leading to the development of new interventions to promote healthy aging. These same interventions may play a role in protecting astronauts and other space explorers on long-duration missions into deep space.

## Figures and Tables

**Table 1:** Genetic variations in antioxidant enzymes and their role in neurological disease susceptibility [16].

Gene	Gene Name	Chromosomal Location	Genetic Variants	Clinical Effects	References
CAT	Catalase	11p13	chr11:34438684C > G/C > T (upstream transcript variant; rs1001179)	Migraine susceptibility	ClinVar, accessed on 12 January 2025
GPX	Glutathione Peroxidases	19p13.3	c.660T > A (SNP; rs713041)	Associated with neurodegeneration and susceptibility to stroke	[17]; ClinVar, accessed on 12 January 2025
GSTM1	Glutathione S-Transferase (mu) M1	1p13.3	GSTM1*0 (homozygous deletion)	Associated with Alzheimer’s disease (AD) pathology	[18,19,20]
GSTT1	Glutathione S-Transferase (theta) T1	22q11.2	GSTT1*0 (homozygous deletion)	AD risk present in Asian populations	[18,19,20]
GSTP1	Glutathione S-Transferase (pi) P1	11q13-qte	c.313A > G (SNP; rs1695)	Associated with AD pathology	[18,19,20]
SOD1	Superoxide Dismutase-1	21q22.11	A4V missense mutation (NeuroX_21:33032096); 16 exonic mutations	ALS pathogenesis	[21]; VarSome, accessed on 12 January 2025
SOD2	Superoxide Dismutase-2	6q25.3	c.47T > C (SNP; rs4880)	Increases Parkinson’s Disease (PD) risk	[22]
PRX	Periaxin	19	2-Cys Prx	Associated with neurodegeneration	ClinVar, accessed on 12 January 2025
HMOX1	Heme Oxygenase 1	22	Length polymorphisms in GT dinucleotide repeats	Associated with vascular diseases	ClinVar, accessed on 12 January 2025

This table summarizes key genetic variants in antioxidant enzymes associated with increased susceptibility to neurological diseases. The highlighted enzymes, including catalase (*CAT*), glutathione peroxidases (*GPX*), glutathione S-transferases (*GSTM1*, *GSTT1*, *GSTP1*), and superoxide dismutases (*SOD1*, *SOD2*), play pivotal roles in mitigating oxidative stress by neutralizing reactive oxygen species (ROS). Genetic variations, such as single nucleotide polymorphisms (SNPs), homozygous deletions, and length polymorphisms, compromise the function of these enzymes, leading to excess ROS accumulation. Elevated ROS levels contribute to oxidative damage to cellular macromolecules, including lipids, proteins, DNA, and RNA. Of particular relevance, the oxidative modification of RNA, including the formation of 8-oxo-guanine, drives RNA–DNA differences (RDDs), which can disrupt transcriptional fidelity and protein synthesis. This process contributes to neuronal dysfunction and degeneration, characteristic of conditions like Alzheimer’s disease (AD), amyotrophic lateral sclerosis (ALS), Parkinson’s disease (PD), and stroke. The table illustrates how specific genetic alterations in antioxidant enzymes amplify oxidative stress, providing a mechanistic link between these variants and the pathogenesis of neurological diseases. Understanding these associations underscores the importance of targeting ROS and RDDs in therapeutic strategies for neurodegenerative disorders.

**Table 2:** Summary of RDD types, mechanisms, and potential consequences.

RDD Type	Mechanism	Affected Nucleotides	Potential Consequences	References
A-to-I editing	Enzymatic deamination by ADAR enzymes	Adenosine to Inosine	Altered protein function, RNA stability, and microRNA binding	[39]
C-to-U editing	Enzymatic deamination by APOBEC enzymes	Cytosine to Uracil	Altered protein function and RNA stability	[40]
m6A editing	Methylation of adenosine	Adenosine	Altered RNA splicing, stability, and translation	[98,99]
Transcriptional errors	Misincorporation, slippage, or template switching by RNA polymerase	Variable	Frameshifts, premature stop codons, altered protein sequence	[100,101]
Oxidative damage	ROS-induced base modification	Primarily Guanine (8-oxoG)	Mispairing during transcription, altered protein sequence, RNA structure disruption	[5,83,102,103]

This table categorizes different types of RNA–DNA differences (RDDs), detailing their underlying mechanisms, the nucleotides affected, and the potential biological consequences. Canonical RNA editing mechanisms, such as A-to-I and C-to-U conversions, are compared alongside non-canonical events like transcriptional errors and oxidative damage, emphasizing their impact on protein function, RNA stability, and cellular processes. References are provided to highlight key studies for each RDD type.

**Table 3:** Cellular mechanisms to counteract RDDs.

Pathway Name	Key Components	Mechanism of Action	References
Nonsense-mediated decay (NMD)	Upf1, Upf2, Upf3	Detects and degrades transcripts with premature stop codons	[121]
No-go decay (NGD)	Dom34, Hbs1	Resolves stalled ribosomes and triggers mRNA cleavage	[122]
Ribosome-associated quality control (RQC)	Ltn1, Rqc1, Cdc48	Degrades incomplete peptides produced during stalled translation	[123]
RNA helicases	Various	Resolve RNA secondary structures induced by oxidative lesions	[124]
Exoribonucleases	Various	Degrade aberrant RNAs	[110]
Unfolded protein response	Various	Copes with misfolded proteins caused by RDDs	[125]

This table outlines the primary cellular pathways that mitigate the effects of RDDs, including nonsense-mediated decay (NMD), no-go decay (NGD), ribosome-associated quality control (RQC), RNA helicases, exoribonucleases, and the unfolded protein response. Each pathway’s key components and mechanisms of action are described, illustrating how cells identify and resolve aberrant RNA transcripts or proteins to maintain homeostasis. References are included to support the described pathways.

**Table 4:** Clinical implications of RDDs.

Disease	Diagnostic Potential	Therapeutic Potential	References
Cancer	RDD profiling for tumor subtyping, prognosis, and treatment response prediction	RDD-based neoantigen discovery for personalized vaccines and adoptive cell therapies; modulation of RNA editing or oxidative stress	[130,131]
Autoimmune diseases	RDD patterns as biomarkers for diagnosis and disease activity monitoring	Modulation of RNA editing or oxidative stress; targeting RDD-derived autoantigens	[132,133,134]
Neurodegenerative diseases	RDD profiling for disease diagnosis and progression monitoring	Antioxidant therapies; enhancement of RNA surveillance mechanisms	[82,83,135,136]

This table explores the role of RDDs in various diseases, including cancer, autoimmune disorders, and neurodegenerative diseases. It highlights the diagnostic potential of RDD profiling for disease subtyping and monitoring, as well as therapeutic strategies that leverage or mitigate RDDs. For each disease, references are provided to link RDDs to diagnostic and treatment innovations.

**Table 5:** Therapeutic strategies targeting RDDs.

Strategy	Mechanism of Action	Potential Applications	References
Antioxidant therapies	Reduce oxidative stress and minimize RDD formation	Prevention and treatment of diseases associated with oxidative damage, including cancer, autoimmune diseases, and neurodegenerative disorders	[16,54]
Modulation of RNA editing	Inhibit or enhance RNA-editing enzymes to alter RDD formation	Cancer immunotherapy (inhibition) and autoimmune disease management (enhancement)	[131,164]
Enhancement of RNA surveillance mechanisms	Boost cellular pathways that degrade or repair aberrant RNAs	Treatment of diseases associated with RDD accumulation, including cancer, autoimmune diseases, and neurodegenerative disorders	[122]
CRISPR-based approaches	Directly correct RDDs at the RNA level	Personalized medicine approaches for correcting disease-causing RDDs	[155]
Hypometabolic state induction	Reduce the metabolic rate, thereby decreasing mitochondrial respiration and ROS production	Protection against oxidative stress in spaceflight, management of acute conditions (e.g., ischemia-reperfusion injury), potential anti-aging intervention	[56,57,58,161,162,163]

This table lists therapeutic approaches aimed at addressing RDD formation or consequences, including antioxidant therapies, modulation of RNA editing, enhancement of RNA surveillance mechanisms, and CRISPR-based interventions. Each strategy’s mechanism of action and potential clinical applications are detailed, emphasizing their relevance to cancer, autoimmune diseases, and neurodegenerative disorders. References are included to substantiate each therapeutic approach.

## References

[1] Brázda V., Kolomazník J., Lýsek J., Bartas M., Fojta M., Šťastný J., Mergny J.-L. (2019). G4Hunter web application: A web server for G-quadruplex prediction. Bioinformatics.

[2] Varshney D., Spiegel J., Zyner K., Tannahill D., Balasubramanian S. (2020). The regulation and functions of DNA and RNA G-quadruplexes. Nat. Rev. Mol. Cell Biol..

[3] Wu S., Jiang L., Lei L., Fu C., Huang J., Hu Y., Dong Y., Chen J., Zeng Q. (2023). Crosstalk between G-quadruplex and ROS. Cell Death Dis..

[4] Springer Nature Switzerland AG (2023). RNA Structure and Function.

[5] Stolc V., Karhanek M., Freund F., Griko Y., Loftus D.J., Ohayon M.M. (2024). Metabolic stress in space: ROS-induced mutations in mice hint at a new path to cancer. Redox Biol..

[6] Poetsch A.R. (2020). The genomics of oxidative DNA damage, repair, and resulting mutagenesis. Comput. Struct. Biotechnol. J..

[7] Li M., Wang I.X., Li Y., Bruzel A., Richards A.L., Toung J.M., Cheung V.G. (2011). Widespread RNA and DNA sequence differences in the human transcriptome. Science.

[8] Paz-Yaacov N., Bazak L., Buchumenski I., Porath H.T., Danan-Gotthold M., Knisbacher B.A., Eisenberg E., Levanon E.Y. (2015). Elevated RNA editing activity is a major contributor to transcriptomic diversity in tumors. Cell Rep..

[9] Lamarck J.-B. (1809). Philosophie Zoologique, ou Exposition des Considérations Relatives à L’histoire Naturelle des Animaux; à la Diversité de Leur Organisation et des 1 Facultés Qu’ils en Obtiennent; aux Causes Physiques qui Maintiennent en eux la vie et Donnent Lieu aux Mouvemens Qu’ils Exécutent; Enfin, à Celles qui Produisent, 2 les unes le Sentiment et les Autres L’intelligence.

[10] Tasaki E., Sakurai H., Nitao M., Matsuura K., Iuchi Y. (2017). Uric acid, an important antioxidant contributing to survival in termites. PLoS ONE.

[11] Ames B.N., Cathcart R., Schwiers E., Hochstein P. (1981). Uric acid provides an antioxidant defense in humans against oxidant- and radical-caused aging and cancer: A hypothesis. Proc. Natl. Acad. Sci. USA.

[12] Johnson R.J., Andrews P., Benner S.A., Oliver W., Theodore E. (2010). Woodward award. The evolution of obesity: Insights from the mid-Miocene. Trans. Am. Clin. Climatol. Assoc..

[13] Conde-Pérezprina J.C., Luna-López A., González-Puertos V.Y., Zenteno-Savín T., León-Galván M.Á., Königsberg M. (2012). DNA MMR systems, microsatellite instability and antioxidant activity variations in two species of wild bats: *Myotis velifer* and *Desmodus rotundus*, as possible factors associated with longevity. Age.

[14] Giani M., Pire C., Martínez-Espinosa R.M. (2024). Bacterioruberin: Biosynthesis, Antioxidant Activity, and Therapeutic Applications in Cancer and Immune Pathologies. Mar. Drugs.

[15] Vauclare P., Wulffelé J., Lacroix F., Servant P., Confalonieri F., Kleman J.-P., Bourgeois D., Timmins J. (2024). Stress-induced nucleoid remodeling in *Deinococcus radiodurans* is associated with major changes in Heat Unstable (HU) protein dynamics. Nucleic Acids Res..

[16] Korczowska-Łącka I., Słowikowski B., Piekut T., Hurła M., Banaszek N., Szymanowicz O., Jagodziński P.P., Kozubski W., Permoda-Pachuta A., Dorszewska J. (2023). Disorders of Endogenous and Exogenous Antioxidants in Neurological Diseases. Antioxidants.

[17] Borchert A., Kalms J., Roth S.R., Rademacher M., Schmidt A., Holzhutter H.G., Kuhn H., Scheerer P. (2018). Crystal structure and functional characterization of selenocysteine-containing glutathione peroxidase 4 suggests an alternative mechanism of peroxide reduction. Biochim. Biophys. Acta Mol. Cell Biol. Lipids.

[18] Bolt H.M., Their R. (2006). Relevance of the deletion polymorphisms of the glutathione S-transferases GSTT1 and GSTM1 in pharmacology and toxicology. Curr. Drug Metab..

[19] Lo H.W., Ali-Osman F. (2007). Genetic Polymorphism and Function of Glutathione S-transferases in Tumor Drug Resistance. Curr. Opin. Pharmacol..

[20] Wang M., Li Y., Lin L., Song G., Deng T. (2016). GSTM1 null genotype and GSTP1 Ile105Val polymorphism are associated with Alzheimer’s disease: A meta-analysis. Mol. Neurobiol..

[21] Andersen P.M., Al-Chalabi A. (2011). Clinical genetics of amyotrophic lateral sclerosis: What do we really know?. Nat. Rev. Neurol..

[22] Singh M., Khan A.J., Singh K. (2008). Association of polymorphism in superoxide dismutase (SOD2) gene with Parkinson’s disease in North Indian population. Indian J. Biochem. Biophys..

[23] Sohn H., Murray D.B., Kuriyama H. (2000). Ultradian oscillation of Saccharomyces cerevisiae during aerobic continuous culture: Hydrogen sulphide mediates population synchrony. Yeast.

[24] Jiang J., Chan A., Ali S., Saha A., Haushalter K.J., Lam W.-L.M., Glasheen M., Parker J., Brenner M., Mahon S.B. (2016). Hydrogen Sulfide—Mechanisms of Toxicity and Development of an Antidote. Sci. Rep..

[25] Ng P.C., Hendry-Hofer T.B., Witeof A.E., Brenner M., Mahon S.B., Boss G.R., Haouzi P., Bebarta V.S. (2019). Hydrogen Sulfide Toxicity: Mechanism of Action, Clinical Presentation, and Countermeasure Development. J. Med. Toxicol..

[26] Munteanu C., Turnea M.A., Rotariu M. (2023). Hydrogen Sulfide: An Emerging Regulator of Oxidative Stress and Cellular Homeostasis—A Comprehensive One-Year Review. Antioxidants.

[27] Bilban M., Haschemi A., Wegiel B., Chin B.Y., Wagner O., Otterbein L.E. (2008). Heme oxygenase and carbon monoxide initiate homeostatic signaling. J. Mol. Med..

[28] Cooper C.E., Brown G.C. (2008). The Inhibition of Mitochondrial Cytochrome Oxidase by the Gases Carbon Monoxide, Nitric Oxide, Hydrogen Cyanide and Hydrogen Sulfide: Chemical Mechanism and Physiological Significance. J. Bioenerg. Biomembr..

[29] Szade A., Szade K., Mahdi M., Józkowicz A. (2021). The role of heme oxygenase-1 in hematopoietic system and its microenvironment. Cell. Mol. Life Sci..

[30] Henrich L., Kiessling I., Steimer M., Frase S., Kaiser S., Schallner N. (2023). Circadian dependency of microglial heme oxygenase-1 expression and inflammation determine neuronal injury in hemorrhagic stroke. J. Inflamm..

[31] Tu B.P., Kudlicki A., Rowicka M., McKnight S.L. (2005). Logic of the yeast metabolic cycle: Temporal compartmentalization of cellular processes. Science.

[32] Tu B.P., McKnight S.L. (2009). Evidence of carbon monoxide-mediated phase advancement of the yeast metabolic cycle. Proc. Natl. Acad. Sci. USA.

[33] Slavov N., Botstein D. (2011). Coupling among growth rate response, metabolic cycle, and cell division cycle in yeast. Mol. Biol. Cell.

[34] Stolc V., Shmygelska A., Griko Y. (2011). Adaptation of organisms by resonance of RNA transcription with the cellular redox cycle. PLoS ONE.

[35] Murray D.B., Lloyd D. (2021). Multiple Rediscoveries and Misconceptions; the Yeast Metabolic Oscillation. Function.

[36] Murray D.B., Beckmann M., Kitano H. (2007). Regulation of Yeast Oscillatory Dynamics. Proc. Natl. Acad. Sci. USA.

[37] Wu F., Du H., Overbey E., Kim J., Makhijani P., Martin N., Lerner C.A., Nguyen K., Baechle J., Valentino T.R. (2024). Single-cell analysis identifies conserved features of immune dysfunction in simulated microgravity and spaceflight. Nat. Commun..

[38] Kumar P., Liu C., Hsu J.W., Chacko S., Minard C., Jahoor F., Sekhar R.V. (2021). Glycine and N-acetylcysteine (GlyNAC) Supplementation in Older Adults Improves Glutathione Deficiency, Oxidative Stress, Mitochondrial Dysfunction, Inflammation, Insulin Resistance, Endothelial Dysfunction, Genotoxicity, Muscle Strength, and Cognition: Results of A Pilot Clinical Trial. Clin. Transl. Med..

[39] Higuchi M., Maas S., Single F.N., Hartner J., Rozov A., Burnashev N., Feldmeyer D., Sprengel R., Seeburg P.H. (2000). Point mutation in an AMPA receptor gene rescues lethality in mice deficient in the RNA-editing enzyme ADAR2. Nature.

[40] Navaratnam N., Bhattacharya S., Fujino T., Patel D., Jarmuz A.L., Scott J. (1995). Evolutionary origins of apoB mRNA editing: Catalysis by a cytidine deaminase that has acquired a novel RNA-binding motif at its active site. Cell.

[41] Cadet J., Davies K.J. (2017). Oxidative DNA damage & repair: An introduction. Free Radic. Biol. Med..

[42] Maizels N., Gray L.T. (2013). The G4 genome. PLoS Genet..

[43] Hahm J.Y., Park J., Jang E.-S., Chi S.W. (2022). 8-Oxoguanine: From oxidative damage to epigenetic and epitranscriptional modification. Exp. Mol. Med..

[44] Schieber M., Chandel N.S. (2014). ROS function in redox signaling and oxidative stress. Curr. Biol..

[45] Mittler R. (2017). ROS Are Good. Trends Plant Sci..

[46] Halliwell B., Adhikary A., Dingfelder M., Dizdaroglu M. (2021). Hydroxyl radical is a significant player in oxidative DNA damage in vivo. Chem. Soc. Rev..

[47] Plante I., West D.W., Weeks J., Risca V.I. (2024). Simulation of Radiation-Induced DNA Damage and Protection by Histones Using the Code RITRACKS. BioTech.

[48] Tian L., Luo Y., Ren J., Zhao C. (2024). The Role of Oxidative Stress in Hypomagnetic Field Effects. Antioxidants.

[49] Kong Q., Lin C.-L.G. (2010). Oxidative damage to RNA: Mechanisms, consequences, and diseases. Cell. Mol. Life Sci..

[50] Turrens J.F. (2003). Mitochondrial formation of reactive oxygen species. J. Physiol..

[51] Wurtmann E.J., Wolin S.L. (2009). RNA under attack: Cellular handling of RNA damage. Crit. Rev. Biochem. Mol. Biol..

[52] D’annibale V., Nardi A.N., Amadei A., D’abramo M. (2021). Theoretical Characterization of the Reduction Potentials of Nucleic Acids in Solution. J. Chem. Theory Comput..

[53] Taylor K.E., Miller L.G., Contreras L.M. (2024). RNA-binding proteins that preferentially interact with 8-oxoG-modified RNAs: Our current understanding. Biochem. Soc. Trans..

[54] Saxena P., Selvaraj K., Khare S.K., Chaudhary N. (2022). Superoxide dismutase as multipotent therapeutic antioxidant enzyme: Role in human diseases. Biotechnol. Lett..

[55] Peng G., Tang Z., Xiang Y., Chen W. (2019). Glutathione peroxidase 4 maintains a stemness phenotype, oxidative homeostasis and regulates biological processes in Panc-1 cancer stem-like cells. Oncol. Rep..

[56] Carey H.V., Andrews M.T., Martin S.L. (2003). Mammalian hibernation: Cellular and molecular responses to depressed metabolism and low temperature. Physiol. Rev..

[57] Storey K.B., Storey J.M. (2004). Metabolic rate depression in animals: Transcriptional and translational controls. Biol. Rev..

[58] Hermes-Lima M., Storey J.M., Storey K.B. (1998). Antioxidant defenses and metabolic depression. The hypothesis of preparation for oxidative stress in land snails. Comp. Biochem. Physiol. Part B Biochem. Mol. Biol..

[59] Barger J.L., Brand M.D., Barnes B.M., Boyer B.B. (2003). Tissue-specific depression of mitochondrial proton leak and substrate oxidation in hibernating arctic ground squirrels. Am. J. Physiol. Integr. Comp. Physiol..

[60] Yamamura Y., Kawamura Y., Oka K., Miura K. (2022). Carcinogenesis resistance in the longest-lived rodent, the naked mole-rat. Cancer Sci..

[61] Podlutsky A.J., Khritankov A.M., Ovodov N.D., Austad S.N. (2005). A new field record for bat longevity. J. Gerontol. Ser. A Biol. Sci. Med. Sci..

[62] Matute J.D., Arias A.A., Wright N.A.M., Wrobel I., Waterhouse C.C.M., Li X.J., Marchal C.C., Stull N.D., Lewis D.B., Steele M. (2009). A new genetic subgroup of chronic granulomatous disease with autosomal recessive mutations in p40 phox and selective defects in neutrophil NADPH oxidase activity. Blood.

[63] Wu Z., Lou Y., Jin W., Liu Y., Lu L., Chen Q., Xie Y., Lu G. (2013). Relationship of the p22phox (CYBA) gene polymorphism C242T with risk of coronary artery disease: A meta-analysis. PLoS ONE.

[64] Stolc V. (1988). Genetic control of blood neutrophil concentration in the rat. Int. J. Immunogenet..

[65] Pleskova S.N., Erofeev A.S., Vaneev A.N., Gorelkin P.V., Bobyk S.Z., Kolmogorov V.S., Bezrukov N.A., Lazarenko E.V. (2023). ROS Production by a Single Neutrophil Cell and Neutrophil Population upon Bacterial Stimulation. Biomedicines.

[66] Tonegawa S. (1983). Somatic generation of antibody diversity. Nature.

[67] Puga I., Cols M., Barra C.M., He B., Cassis L., Gentile M., Comerma L., Chorny A., Shan M., Xu W. (2011). B cell–helper neutrophils stimulate the diversification and production of immunoglobulin in the marginal zone of the spleen. Nat. Immunol..

[68] Sontz P.A., Mui T.P., Fuss J.O., Tainer J.A., Barton J.K. (2012). DNA charge transport as a first step in coordinating the detection of lesions by repair proteins. Proc. Natl. Acad. Sci. USA.

[69] Amin M., Brooks B.R. (2024). The oxidation of the [4Fe-4S] cluster of DNA primase alters the binding energies with DNA and RNA primers. Biophys. J..

[70] O’brien E., Holt M.E., Thompson M.K., Salay L.E., Ehlinger A.C., Chazin W.J., Barton J.K. (2017). The [4Fe4S] cluster of human DNA primase functions as a redox switch using DNA charge transport. Science.

[71] Amariei C., Machne R., Stolc V., Soga T., Tomita M., Murray D.B. (2014). Time resolved DNA occupancy dynamics during the respiratory oscillation uncover a global reset point in the yeast growth program. Microb. Cell.

[72] Bochman M.L., Schwacha A. (2009). The Mcm complex: Unwinding the mechanism of a replicative helicase. Microbiol. Mol. Biol. Rev..

[73] Seo Y.-S., Kang Y.-H. (2018). The Human Replicative Helicase, the CMG Complex, as a Target for Anti-cancer Therapy. Front. Mol. Biosci..

[74] Juan C.A., de la Lastra J.M.P., Plou F.J., Pérez-Lebeña E. (2021). The Chemistry of Reactive Oxygen Species (ROS) Revisited: Outlining Their Role in Biological Macromolecules (DNA, Lipids and Proteins) and Induced Pathologies. Int. J. Mol. Sci..

[75] Kjær L.K., Cejvanovic V., Henriksen T., Petersen K.M., Hansen T., Pedersen O., Christensen C.K., Torp-Pedersen C., Gerds T.A., Brandslund I. (2017). Cardiovascular and All-Cause Mortality Risk Associated with Urinary Excretion of 8-oxoGuo, a Biomarker for RNA Oxidation, in Patients with Type 2 Diabetes: A Prospective Cohort Study. Diabetes Care.

[76] Rahman I., Adcock I.M. (2006). Oxidative stress and redox regulation of lung inflammation in COPD. Eur. Respir. J..

[77] MacNee W. (2001). Oxidative stress and lung inflammation in airways disease. Eur. J. Pharmacol..

[78] Barnes P.J. (2022). Oxidative Stress in Chronic Obstructive Pulmonary Disease. Antioxidants.

[79] Gastelum S., Michael A.F., Bolger T.A. (2023). *Saccharomyces cerevisiae* as a research tool for RNA-mediated human disease. Wiley Interdiscip. Rev. RNA.

[80] Back P., Braeckman B.P., Matthijssens F. (2012). ROS in aging *Caenorhabditis elegans*: Damage or signaling?. Oxidative Med. Cell. Longev..

[81] von Zglinicki T. (2002). Oxidative stress shortens telomeres. Trends Biochem. Sci..

[82] Fischer L.R., Li Y., Asress S.A., Jones D.P., Glass J.D. (2012). Absence of SOD1 leads to oxidative stress in peripheral nerve and causes a progressive distal motor axonopathy. Exp. Neurol..

[83] Nunomura A., Perry G., Pappolla M.A., Wade R., Hirai K., Chiba S., Smith M.A. (1999). RNA oxidation is a prominent feature of vulnerable neurons in Alzheimer’s disease. J. Neurosci..

[84] Gámez-Valero A., Guisado-Corcoll A., Herrero-Lorenzo M., Solaguren-Beascoa M., Martí E. (2020). Non-Coding RNAs as Sensors of Oxidative Stress in Neurodegenerative Diseases. Antioxidants.

[85] Panatta E., Zampieri C., Melino G., Amelio I. (2021). Understanding p53 tumour suppressor network. Biol. Direct.

[86] Emre Y., Nübel T. (2010). Uncoupling protein UCP2: When mitochondrial activity meets immunity. FEBS Lett..

[87] Li W., Zhang C., Jackson K., Shen X., Jin R., Li G., Kevil C.G., Gu X., Shi R., Zhao Y. (2015). UCP2 knockout suppresses mouse skin carcinogenesis. Cancer Prev. Res..

[88] Mihaljevic O., Zivancevic-Simonovic S., Jovanovic D., Drakulic S.M., Vukajlovic J.T., Markovic A., Pirkovic M.S., Srejovic I., Jakovljevic V., Milosevic-Djordjevic O. (2023). Oxidative stress and DNA damage in critically ill patients with sepsis. Mutat. Res. Toxicol. Environ. Mutagen..

[89] Cooke M.S., Evans M.D., Dizdaroglu M., Lunec J. (2003). Oxidative DNA damage: Mechanisms, mutation, and disease. FASEB J..

[90] Sorrentino Z.A., Vijayaraghavan N., Gorion K.-M., Riffe C.J., Strang K.H., Caldwell J., Giasson B.I. (2018). Physiological C-terminal truncation of α-synuclein potentiates the prion-like formation of pathological inclusions. J. Biol. Chem..

[91] Wheeler H.B., Madrigal A.A., Chaim I.A. (2024). Mapping the future of oxidative RNA damage in neurodegeneration: Rethinking the status quo with new tools. Proc. Natl. Acad. Sci. USA.

[92] Kumar D., Abdulovic A.L., Viberg J., Nilsson A.K., Kunkel T.A., Chabes A. (2011). Mechanisms of mutagenesis in vivo due to imbalanced dNTP pools. Nucleic Acids Res..

[93] Ragu S., Faye G., Iraqui I., Masurel-Heneman A., Kolodner R.D., Huang M.-E. (2007). Oxygen metabolism and reactive oxygen species cause chromosomal rearrangements and cell death. Proc. Natl. Acad. Sci. USA.

[94] Iraqui I., Kienda G., Soeur J., Faye G., Baldacci G., Kolodner R.D., Huang M.-E. (2009). Peroxiredoxin Tsa1 is the key peroxidase suppressing genome instability and protecting against cell death in *Saccharomyces cerevisiae*. PLoS Genet..

[95] Degtyareva N.P., Chen L., Mieczkowski P., Petes T.D., Doetsch P.W. (2008). Chronic oxidative DNA damage due to DNA repair defects causes chromosomal instability in *Saccharomyces cerevisiae*. Mol. Cell. Biol..

[96] Evert B.A., Salmon T.B., Song B., Liu J., Siede W., Doetsch P.W. (2004). Spontaneous DNA damage in *Saccharomyces cerevisiae* elicits phenotypic properties similar to cancer cells. J. Biol. Chem..

[97] Kumar D., Viberg J., Nilsson A.K., Chabes A. (2010). Highly mutagenic and severely imbalanced dNTP pools can escape detection by the S-phase checkpoint. Nucleic Acids Res..

[98] Dominissini D., Moshitch-Moshkovitz S., Schwartz S., Salmon-Divon M., Ungar L., Osenberg S., Cesarkas K., Jacob-Hirsch J., Amariglio N., Kupiec M. (2012). Topology of the human and mouse m6A RNA methylomes revealed by m6A-seq. Nature.

[99] Meyer K.D., Saletore Y., Zumbo P., Elemento O., Mason C.E., Jaffrey S.R. (2012). Comprehensive analysis of mRNA methylation reveals enrichment in 3′ UTRs and near stop codons. Cell.

[100] Atkins J.F., Loughran G., Bhatt P.R., Firth A.E., Baranov P.V. (2016). Ribosomal frameshifting and transcriptional slippage: From genetic steganography and cryptography to adventitious use. Nucleic Acids Res..

[101] Xu L., Wang W., Chong J., Shin J.H., Xu J., Wang D. (2015). RNA polymerase II transcriptional fidelity control and its functional interplay with DNA modifications. Crit. Rev. Biochem. Mol. Biol..

[102] Tanaka M., Chock P.B., Stadtman E.R. (2007). Oxidative RNA damage and lifespan in yeast. Mol. Biol. Cell.

[103] Simms C.L., Zaher H.S. (2016). Quality control of chemically damaged RNA. Cell. Mol. Life Sci..

[104] Stolc V., Altman S. (1997). Rpp1, an essential protein subunit of nuclear RNase P required for processing of precursor tRNA and 35S precursor rRNA in *Saccharomyces cerevisiae*. Genes Dev..

[105] Samanta M.P., Tongprasit W., Sethi H., Chin C.-S., Stolc V. (2006). Global identification of noncoding RNAs in *Saccharomyces cerevisiae* by modulating an essential RNA processing pathway. Proc. Natl. Acad. Sci. USA.

[106] Guerrier-Takada C., Gardiner K., Marsh T., Pace N., Altman S. (1983). The RNA moiety of ribonuclease P is the catalytic subunit of the enzyme. Cell.

[107] Jarrous N., Liu F. (2023). Human RNase P: Overview of a ribonuclease of interrelated molecular networks and gene-targeting systems. RNA.

[108] Jiang M., Wang H., Liu Z., Lin L., Wang L., Xie M., Li D., Zhang J., Zhang R. (2020). Endoplasmic reticulum stress-dependent activation of iNOS/NO-NF-κB signaling and NLRP3 inflammasome contributes to endothelial inflammation and apoptosis associated with microgravity. FASEB J..

[109] Cech T.R. (2018). A Lifelong Passion for All Things Ribonucleic. Cell.

[110] Houseley J., Tollervey D. (2009). The many pathways of RNA degradation. Cell.

[111] Beckman K.B., Ames B.N. (1998). The Free Radical Theory of Aging Matures. Physiol. Rev..

[112] Speakman J.R. (2005). Body Size, Energy Metabolism and Lifespan. J. Exp. Biol..

[113] Perez V.I., Bokov A., Van Remmen H., Mele J., Ran Q., Ikeno Y., Richardson A. (2009). Is the Oxidative Stress Theory of Aging Dead?. Biochim. Biophys. Acta Gen. Subj..

[114] Vaghf A., Khansarinejad B., Ghaznavi-Rad E., Mondanizadeh M. (2022). The role of microRNAs in diseases and related signaling pathways. Mol. Biol. Rep..

[115] Cui L., Ma R., Cai J., Guo C., Chen Z., Yao L., Wang Y., Fan R., Wang X., Shi Y. (2022). RNA modifications: Importance in immune cell biology and related diseases. Signal Transduct. Target. Ther..

[116] Yuan J., Xu L., Bao H.-J., Wang J.-L., Zhao Y., Chen S. (2023). Biological roles of A-to-I editing: Implications in innate immunity, cell death, and cancer immunotherapy. J. Exp. Clin. Cancer Res..

[117] Boccitto M., Wolin S.L. (2019). Ro60 and Y RNAs: Structure, functions, and roles in autoimmunity. Crit. Rev. Biochem. Mol. Biol..

[118] Shay J.W., Wright W.E. (2019). Telomeres and telomerase: Three decades of progress. Nat. Rev. Genet..

[119] Hemagirri M., Sasidharan S. (2022). Biology of aging: Oxidative stress and RNA oxidation. Mol. Biol. Rep..

[120] Asche-Godin S.L., A Graham Z., Israel A., Harlow L.M., Huang W., Wang Z., Brotto M., Mobbs C., Cardozo C.P., Ko F.C. (2022). RNA-sequencing Reveals a Gene Expression Signature in Skeletal Muscle of a Mouse Model of Age-associated Postoperative Functional Decline. J. Gerontol. Biol. Sci..

[121] Chang Y.F., Imam J.S., Wilkinson M.F. (2007). Nonsense-mediated mRNA Decay: A Mechanistic Perspective. Annu. Rev. Biochem..

[122] Doma M.K., Parker R. (2007). RNA quality control in eukaryotes. Cell.

[123] Defenouillère Q., Zhang E., Namane A., Mouaikel J., Jacquier A., Fromont-Racine M. (2016). Rqc1 and Ltn1 Prevent C-terminal Alanine-Threonine Tail (CAT-tail)-induced Protein Aggregation by Efficient Recruitment of Cdc48 on Stalled 60S Subunits. J. Biol. Chem..

[124] Jankowsky E., Fairman M.E. (2007). RNA Helicases—One Fold for Many Functions. Curr. Opin. Struc. Biol..

[125] Hetz C., Papa F.R. (2018). The Unfolded Protein Response and Cell Fate Control. Mol. Cell..

[126] Behera A., Panigrahi G.K., Sahoo A. (2024). Nonsense-Mediated mRNA Decay in Human Health and Diseases: Current Understanding, Regulatory Mechanisms and Future Perspectives. Mol. Biotechnol..

[127] Yan L.L., Simms C.L., McLoughlin F., Vierstra R.D., Zaher H.S. (2019). Oxidation and alkylation stresses activate ribosome-quality control. Nat. Commun..

[128] Wang I.X., Grunseich C., Chung Y.G., Kwak H., Ramrattan G., Zhu Z., Cheung V.G. (2016). RNA-DNA sequence differences in Saccharomyces cerevisiae. Genome Res..

[129] Hashimoto S., Noguchi E., Bando H., Miyadera H., Morii W., Nakamura T., Hara H. (2021). Neoantigen prediction in human breast cancer using RNA sequencing data. Cancer Sci..

[130] Toung J.M., Morley M., Li M., Cheung V.G. (2014). RNA editing as a source of tumor-specific antigenic diversity in immunotherapy. PLoS ONE.

[131] Han L., Diao L., Yu S., Xu X., Li J., Zhang R., Yang Y., Werner H.M., Eterovic A.K., Yuan Y. (2015). The Genomic Landscape and Clinical Relevance of A-to-I RNA Editing in Human Cancers. Cancer Cell.

[132] Peng M., Mo Y., Wang Y., Wu P., Zhang Y., Xiong F., Guo C., Wu X., Li Y., Li X. (2019). Neoantigen vaccine: An emerging tumor immunotherapy. Mol. Cancer.

[133] Li Q., Gloudemans M.J., Geisinger J.M., Fan B., Aguet F., Sun T., Ramaswami G., Li Y.I., Ma J.-B., Pritchard J.K. (2022). RNA editing underlies genetic risk of common inflammatory diseases. Nature.

[134] Blanco-Melo D., Nilsson-Payant B.E., Liu W.C., Uhl S., Hoagland D., Møller R., Jordan T.X., Oishi K., Panis M., Sachs D. (2020). Imbalanced Host Response to SARS-CoV-2 Drives Development of COVID-19. Cell.

[135] Jorgensen A., Brandslund I., Ellervik C., Henriksen T., Weimann A., Andersen P.K., Poulsen H.E. (2023). Specific prediction of mortality by oxidative stress-induced damage to RNA vs. DNA in humans. Aging Cell.

[136] Jorgensen A., Brandslund I., Ellervik C., Henriksen T., Weimann A., Andersen M.P., Torp-Pedersen C., Andersen P.K., Jorgensen M.B., Poulsen H.E. (2024). Oxidative Stress-Induced Damage to RNA and DNA and Mortality in Individuals with Psychiatric Illness. JAMA Psychiatry.

[137] Zhou C., Wei Z., Zhang L., Yang Z., Liu Q. (2020). Systematically Characterizing A-to-I RNA Editing Neoantigens in Cancer. Front. Oncol..

[138] Jiang T., Shi T., Zhang H., Hu J., Song Y., Wei J., Ren S., Zhou C. (2019). Tumor neoantigens: From basic research to clinical applications. J. Hematol. Oncol..

[139] Zeng H., Gifford D.K. (2019). Quantification of Uncertainty in Peptide-MHC Binding Prediction Improves High-Affinity Peptide Design. Cell Syst..

[140] Fritsch E.F., Ott P.A. (2024). Personalized Cancer Vaccines Directed against Tumor Mutations: Building Evidence from Mice to Humans. Cancer Res..

[141] Pang Z., Lu M.-M., Zhang Y., Gao Y., Bai J.-J., Gu J.-Y., Xie L., Wu W.-Z. (2023). Neoantigen-targeted TCR-engineered T cell immunotherapy: Current advances and challenges. Biomark. Res..

[142] Time (2023). A Melanoma Vaccine Showed Promising Results in a New Study. TIME.

[143] Goodchild A., Nopper N., King A., Doan T., Tanudji M., Arndt G.M., Poidinger M., Rivory L.P., Passioura T. (2009). Sequence determinants of innate immune activation by short interfering RNAs. BMC Immunol..

[144] Wang T., Song D., Li X., Luo Y., Yang D., Liu X., Kong X., Xing Y., Bi S., Zhang Y. (2024). MiR-574-5p activates human TLR8 to promote autoimmune signaling and lupus. Cell Commun. Signal..

[145] Nislow C., Lee A.Y., Allen P.L., Giaever G., Smith A., Gebbia M., Stodieck L.S., Hammond J.S., Birdsall H.H., Hammond T.G. (2015). Genes required for survival in microgravity revealed by genome-wide yeast deletion collections cultured during spaceflight. BioMed Res. Int..

[146] Dugbartey G.J. (2024). Cellular and molecular mechanisms of cell damage and cell death in ischemia–reperfusion injury in organ transplantation. Mol. Biol. Rep..

[147] Wang M., Liu Y., Liang Y., Naruse K., Takahashi K. (2021). Systematic understanding of pathophysiological mechanisms of oxidative stress-related conditions—Diabetes mellitus, cardiovascular diseases, and ischemia–reperfusion injury. Front. Cardiovasc. Med..

[148] Che W., Asahi M., Takahashi M., Kaneto H., Okado A., Higashiyama S., Taniguchi N. (1997). Selective Induction of Heparin-binding Epidermal Growth Factor-like Growth Factor by Methylglyoxal and 3-Deoxyglucosone in Rat Aortic Smooth Muscle Cells. J. Biol. Chem..

[149] Iqbal M.J., Kabeer A., Abbas Z., Siddiqui H.A., Calina D., Sharifi-Rad J., Cho W.C. (2024). Interplay of oxidative stress, cellular communication and signaling pathways in cancer. Cell Commun. Signal..

[150] Panda S., Chatterjee O., Mukherjee G., Chatterjee S., Chatterjee S., Chattopadhyay S. (2023). Human Diseases Induced by Oxidative Damage in DNA. Nucleic Acid Biology and Its Application in Human Diseases.

[151] Ezerina D., Takano Y., Hanaoka K., Urano Y., Dick T.P. (2018). N-Acetyl Cysteine Functions as A Fast-acting Antioxidant by Triggering Intracellular H2S and Sulfane Sulfur Production. Cell Chem. Biol..

[152] Roberson S.W., Nwosu S., Collar E.M., Kiehl A.L., Harrison F.E., Bastarache J., Wilson J.E., Mart M.F., Sevransky J.E., Ely E.W. (2023). Association of Vitamin C, Thiamine, and Hydrocortisone Infusion with Long-term Cognitive, Psychological, and Functional Outcomes in Sepsis Survivors: A Secondary Analysis of the Vitamin C, Thiamine, and Steroids in Sepsis Randomized Clinical Trial. JAMA Netw. Open.

[153] Amrein K., Oudemans-van Straaten H.M., Berger M.M. (2018). Vitamin therapy in critically ill patients: Focus on thiamine, vitamin C, and vitamin D. Intensive Care Med..

[154] Costa N.A., Pereira A.G., Sugizaki C.S.A., Vieira N.M., Garcia L.R., de Paiva S.A.R., Zornoff L.A.M., Azevedo P.S., Polegato B.F., Minicucci M.F. (2022). Insights into Thiamine Supplementation in Patients with Septic Shock. Front. Med..

[155] Cox D.B.T., Platt R.J., Zhang F. (2015). Therapeutic genome editing: Prospects and challenges. Nat. Med..

[156] Cox D.B.T., Gootenberg J.S., Abudayyeh O.O., Franklin B., Kellner M.J., Joung J., Zhang F. (2017). RNA editing with CRISPR-Cas13. Science.

[157] Bergendi L., Beneš L., Ďuračková Z., Ferenčik M. (1999). Chemistry, physiology and pathology of free radicals. Life Sci..

[158] Esteller M. (2011). Non-coding RNAs in human disease. Nat. Rev. Genet..

[159] Baird B.J., Dickey J.S., Nakamura A.J., Redon C.E., Parekh P., Griko Y.V., Aziz K., Georgakilas A.G., Bonner W.M., Martin O.A. (2011). Hypothermia postpones DNA damage repair in irradiated cells and protects against cell killing. Mutat. Res..

[160] Griko Y., Loftus D., Stolc V. (2024). Metabolic Suppression: A Promising Solution to Unlock the Future of Space Travel. J. Tour. Hosp..

[161] Ma S., Han C., Wang D., Xie Z., Liu J., Li Z. (2022). Synthetic torpor: A paradigm shift in clinical practice?. Front. Pharmacol..

[162] Hainsworth A.H., Drinkhill M.J. (2023). Therapeutic potential of hypometabolism for protection against ischemia-reperfusion injury. Pharmacol. Ther..

[163] Blanc V., Davidson N.O. (2003). APOBEC-1-mediated RNA editing. Wiley Interdiscip. Rev. Syst. Biol. Med..

[164] Boutilier R.G., St-Pierre J. (2000). Surviving hypoxia without really dying. Comp. Biochem. Physiol. A Mol. Integr. Physiol..

[165] Kiani L. (2024). Genetic protection against Alzheimer disease. Nat. Rev. Neurol..

[166] Tesi N., van der Lee S., Hulsman M., van Schoor N.M., Huisman M., Pijnenburg Y., van der Flier W.M., Reinders M., Holstege H. (2024). Cognitively healthy centenarians are genetically protected against Alzheimer’s disease. Alzheimer’s Dement..

